# Therapeutic application of quercetin in aging-related diseases: SIRT1 as a potential mechanism

**DOI:** 10.3389/fimmu.2022.943321

**Published:** 2022-07-22

**Authors:** Zhifu Cui, Xingtao Zhao, Felix Kwame Amevor, Xiaxia Du, Yan Wang, Diyan Li, Gang Shu, Yaofu Tian, Xiaoling Zhao

**Affiliations:** ^1^ Farm Animal Genetic Resources Exploration and Innovation Key Laboratory of Sichuan Province, Sichuan Agricultural University, Chengdu, China; ^2^ State Key Laboratory of Southwestern Chinese Medicine Resources, Ministry of Education, Chengdu University of Traditional Chinese Medicine, Chengdu, China; ^3^ Department of Basic Veterinary Medicine, Sichuan Agricultural University, Chengdu, China

**Keywords:** quercetin, aging-related diseases, sirtuin 1, oxidative stress, mitochondrial dysfunction, inflammatory response

## Abstract

Quercetin, a naturally non-toxic flavonoid within the safe dose range with antioxidant, anti-apoptotic and anti-inflammatory properties, plays an important role in the treatment of aging-related diseases. Sirtuin 1 (SIRT1), a member of NAD^+^-dependent deacetylase enzyme family, is extensively explored as a potential therapeutic target for attenuating aging-induced disorders. SIRT1 possess beneficial effects against aging-related diseases such as Alzheimer’s disease (AD), Parkinson’s disease (PD), Huntington’s disease (HD), Depression, Osteoporosis, Myocardial ischemia (M/I) and reperfusion (MI/R), Atherosclerosis (AS), and Diabetes. Previous studies have reported that aging increases tissue susceptibility, whereas, SIRT1 regulates cellular senescence and multiple aging-related cellular processes, including SIRT1/Keap1/Nrf2/HO-1 and SIRTI/PI3K/Akt/GSK-3β mediated oxidative stress, SIRT1/NF-κB and SIRT1/NLRP3 regulated inflammatory response, SIRT1/PGC1α/eIF2α/ATF4/CHOP and SIRT1/PKD1/CREB controlled phosphorylation, SIRT1-PINK1-Parkin mediated mitochondrial damage, SIRT1/FoxO mediated autophagy, and SIRT1/FoxG1/CREB/BDNF/Trkβ-catenin mediated neuroprotective effects. In this review, we summarized the role of SIRT1 in the improvement of the attenuation effect of quercetin on aging-related diseases and the relationship between relevant signaling pathways regulated by SIRT1. Moreover, the functional regulation of quercetin in aging-related markers such as oxidative stress, inflammatory response, mitochondrial function, autophagy and apoptosis through SIRT1 was discussed. Finally, the prospects of an extracellular vesicles (EVs) as quercetin loading and delivery, and SIRT1-mediated EVs as signal carriers for treating aging-related diseases, as well as discussed the ferroptosis alleviation effects of quercetin to protect against aging-related disease *via* activating SIRT1. Generally, SIRT1 may serve as a promising therapeutic target in the treatment of aging-related diseases *via* inhibiting oxidative stress, reducing inflammatory responses, and restoring mitochondrial dysfunction.

**Graphical Abstract:**
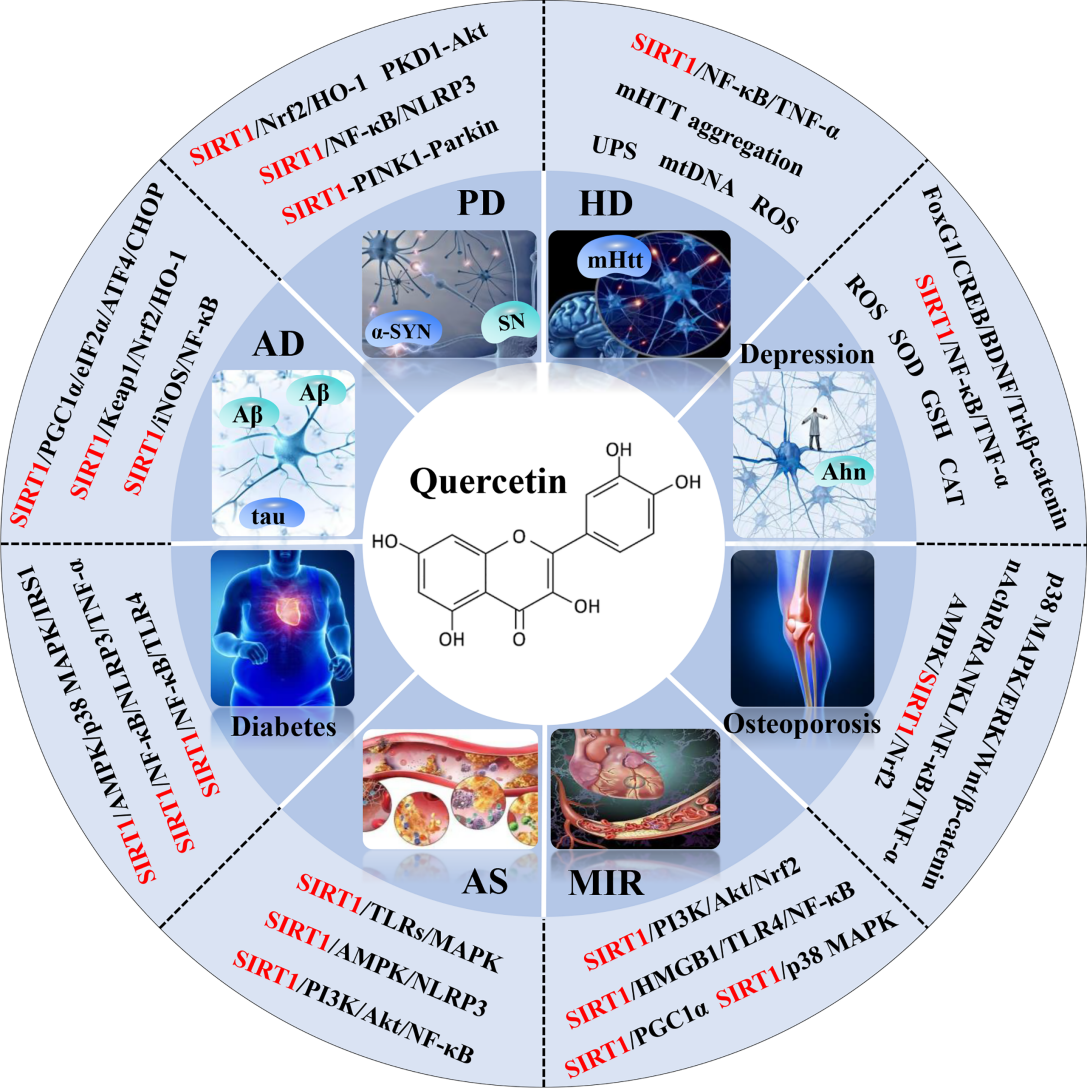


## Highlights:

1. Quercetin, a naturally non-toxic flavonoid within the safe dose range with antioxidant, anti-apoptotic, and anti-inflammatory properties, plays an important role in the treatment of aging-related diseases.
2. Quercetin exerts neuroprotective effects against chronic aging-related diseases *via* targeting SIRT1 to regulate cellular senescence and multiple aging-related cellular processes such as SIRT1/Keap1/Nrf2/HO-1 and PI3K/Akt/GSK-3β mediated oxidative stress, SIRT1/NF-κB mediated inflammatory response, SIRT1/PGC1α/eIF2α/ATF4/CHOP mediated mitochondrial damage, and SIRT1/FoxO mediated autophagy.
3. Studies on the preventive and therapeutic effects and clinical application of natural SIRT1 activator or synthetic SIRT1 activator on aging-related diseases could provide a strong foundation and basis for investigating further potential target drugs to attenuate aging-related diseases.

## 1 Introduction

Aging in both animals and humans is positively correlated with a decline in the physiological functions, thereby increasing the occurrence of aging-related diseases; such as neurodegenerative diseases (NDS) (1) including Alzheimer’s disease (AD) (2), Parkinson’s disease (PD) (3), Huntington’s disease (HD) (4), and Depression (5); as well as other related diseases such as Osteoporosis (6), Myocardial ischemia (M/I) and reperfusion (MI/R) (7), Atherosclerosis (AS) (8), and Diabetes (9). A study reported that aging-related NDS caused significant morbidity and economic burden of approximately $25 billion annually in the USA (10). Therefore, several researchers engaged in studies to explore the molecular mechanism of various aging related diseases.

Chronological aging cause subtle changes in the neuronal structure and function of specific neuronal circuits, resulting in significant decrease in dopamine receptors in the striatum and reactive microglia and astrocytes, and the overactivation of proinflammatory microglia which triggers chronic inflammation (11). The production and accumulation of free radicals causes oxidative stress which increases the production of proinflammatory cytokines, leading to neuronal cell death and glial cell activation (12, 13). However, Sirtuin 1 (SIRT1) regulates the expression of proinflammatory cytokines such as tumor necrosis factor (TNF), interleukin-1 β (IL-1β), and interferon γ (IFN-γ) during microglial activation (14), as well as alleviates degeneration of dopaminergic neurons (15).

Sirtuin (SIRT), a member of the NAD^+^-dependent deacetylase enzyme family, regulates various cellular targets and functions. SIRT1 is the most widely studied Sirtuin in mammals and is considered as a potential therapeutic target for aging-associated disorders (16–18). SIRT1 activation was reported to attenuate other age-associated disorders such as NDS (19–21). Several aging-related markers such as neuroinflammation, oxidative stress, activation of glial cell, inhibition of adaptive neuroplasticity, dysregulation of neuronal Ca^2+^ homeostasis, mitochondrial dysfunction, and cellular senescence elevate the risk of aging-related diseases (22). Studies have indicated that SIRT1 silencing intensify the formation of aging-related inclusions containing alpha-synuclein, increase the number of age-dependent degeneration of dopaminergic neurons, which intensify the progression of related diseases (23). Moreover, excessive production of free radicals causes cell damage, leading to oxidative and nitrosative stress, which disrupts the immune function and hence, induces series of aging-related diseases. SIRT1 modulates the production and accumulation of reactive oxygen species (ROS) *in vitro* and *in vivo*, thereby attenuating oxidative stress associated with neurodegeneration (15, 24).

ROS accumulation cause mitochondrial dysfunction and cell death due to excessive inflammation (25), this allows the development of aging-related diseases (26). However, SIRT1 promotes mitochondrial function and regulates mitochondrial homeostasis. Studies have indicated that overexpression of SIRT1 can effectively inhibit cell death, promote cell survival, and prolong the lifespan of cells (27). However, available strategies such as chemical drugs to treat diseases are very expensive, less clinically effective, and have obvious toxic effects. Therefore, researchers are currently studying to develop potent, cheap, safe, and more effective natural alternatives or novel targets to attenuate aging-induced diseases.

**Figure 1:**
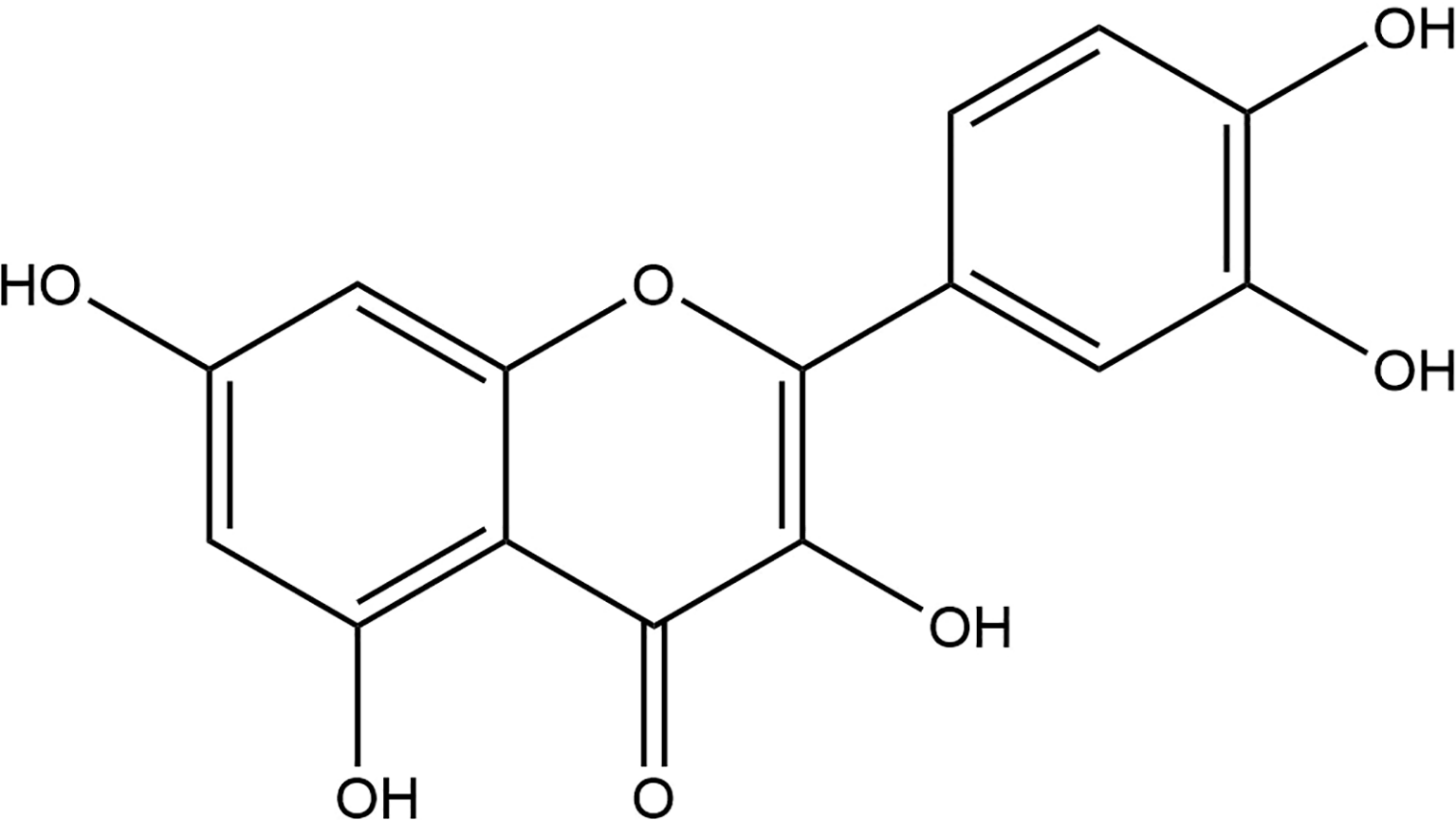
Chemical formula of quercetin.

Quercetin (2-(3,4-dihydroxy phenyl)-3,5,7-trihydroxy-4H-1-benzopyran-4-one) (**Figure 1**) is one of the major naturally nontoxic flavonoids which is widely found in fruits (grapes, peaches) and vegetables (onions, garlic). Quercetin is a lipophilic compound that could be absorbed by simple diffusion across the intestinal membrane, however, it is ingested primarily as a glycoside, which is converted to a glycoside ligand in the intestine, and then release by absorption into the intestinal epithelium through the action of β-glycosidase, and both the intestinal and oral bacteria are involved in this enzymatic hydrolysis. Studies have shown that poor oral bioavailability of single doses (28–30). The poor aqueous solubility of quercetin (~1 mg/mL in water, ~5.5 mg/mL in simulated gastric fluid, and ~28.9 mg/mL in simulated intestinal fluid) and its instability in the physiological media have limited its application in pharmacology (31, 32), great efforts have been made in its drug delivery systems to address the problem of limited application (33, 34).

Quercetin plays many beneficial roles such as antioxidant (35), anti-inflammatory (36), anti-apoptotic (37) and other biological activities (38) in neuroprotection (39). Reports have indicated that quercetin contain anti-aging properties (31, 40–43). However, the mechanisms through which quercetin exert these functions are complex and the signaling pathways are intertwined. Therefore, our aim in this review was to clarify how quercetin targets SIRT1 to prevent aging-related diseases, elucidate the relationships between the relevant signaling pathways, provide the possible targets and the theoretical basis for quercetin to serve as an effective drug for aging-related diseases.

## 2 Effects of quercetin on neurodegenerative diseases

### 2.1 Alzheimer’s disease (AD)

Alzheimer’s disease (AD) is a neurodegenerative disorder, characterized by learning and memory dysfunction at an early stage and eventually evolves into a cognitive disorder. Due to the progression of the disease and limited treatment options, AD has become one of the greatest threats to the modern population, accounting for approximately 45 million patients worldwide. Studies have predicted that by the year 2050, approximately 150 million people may suffer from AD (2).

The mechanisms of AD are related to the deposition of β-amyloid (Aβ) peptides and intracellular neurofibrillary tangles consisting of hyperphosphorylated tau protein, which are important characteristics of AD and can lead to serial neuronal loss and brain atrophy (44). SIRT1 regulates the expression of Aβ (45) and tau (46), which are associated with AD. A study using a three-transgenic AD (3XTG-AD) mice model, reported that quercetin (100 mg/kg) significantly improved the biomarkers of neurodegeneration and cognitive and emotional deficits (47).

#### 2.1.1 Regulation of oxidative stress

In addition to its direct toxic effects on neurons, Aβ increases the sensitivity of neurons to harmful factors such as free radicals and oxidative stress, which play important role during AD. Quercetin supplementation reduce microglia aggregation around amyloid plaques in AD mice (48) through various excess protein pathways (49). Activation of SIRT1 was reported to counteract oxidative stress induced by Aβ aggregation (50). In addition, Yu et al. reported that quercetin regulates SIRT1/Nrf2/HO-1 pathway thereby exerting neuroprotective effects against AD (51). In HT22 hippocampal neurons, quercetin was reported to show a neuroprotective effect by promoting phosphoinositide 3-kinase (PI3K)/Akt which could downregulate glycogen synthase kinase 3beta (GSK-3β) activity (52), thereby reducing oxidative stress-mediated neuronal death.

#### 2.1.2 Regulation of inflammatory response

Aβ promotes the activation of microglia and the release of inflammatory cytokines. Chronic inflammation is associated with age-related Alzheimer’s disease. Studies have shown that quercetin exerts an anti-neuroinflammatory effects on LPS-activated BV-2 microglial cells (53), by attenuating the production of inflammatory mediators, including nitric oxide (NO) and TNF, as well as reduce the level of inducible NO synthase (iNOS) (54). Quercetin reduces ROS production by promoting the activity of SIRT1 and enhancing the anti-inflammatory activity of NF-κB acetylation in the neural and glial cells, thereby reducing neuronal cell death (55).

#### 2.1.3 Regulation of mitochondrial function

Mitochondria are organelles responsible for ATP production, calcium regulation, and regulation of redox homeostasis. Mitochondrial dysfunction is regarded as the main causative factor of the pathogenesis aging-related AD (56), as a result could inflict damage to neurons, microglia, and astrocytes (57). Microglia phagocytose Aβ and the surrounding Aβ plaques promote the synaptic and neuronal loss in the chronic AD process in the mitochondrial dysfunction and hence, increase LAMP1 immunoreactivity, thereby preventing the diffusion of soluble amyloid into the surrounding thin-walled brain (58). In addition, quercetin was found to improve the cognitive function in the APPswe/PS1De9 transgenic mice model during chronic AD, by reducing mitochondrial dysfunction *via* the activation of AMP-activated protein kinase (AMPK) (59). Moreover, SIRT1 regulates mitochondrial biogenesis and function by directly controlling the activity of proliferator-activated receptor gamma coactivator 1alpha (PGC-1α) through phosphorylation and deacetylation (60), whereas quercetin cause a reduction in the phosphorylation of the eukaryotic initiation factor 2 alpha (eIF2α), as well as activate the expression of transcription factor 4 (ATF4) through the GADD34 induction in the brain, leading to memory improvement in aged mice and delayed memory deterioration at the early stage of APP23 AD model mice (61).

#### 2.1.4 Regulation of autophagy function

Autophagy is the main mechanism underlying the onset and progression of AD (62). FoxO proteins, key substrates of SIRT1, are prominent and necessary factors in the formation of memory and cognitive function (63). Studies in a drosophila AD model have shown that quercetin significantly alleviate chronic AD by restoring the expression of cell cycle protein including cell cycle proteins B located in the FoxO signaling pathway perturbed by Aβ accumulation (64).

**Figure 2:**
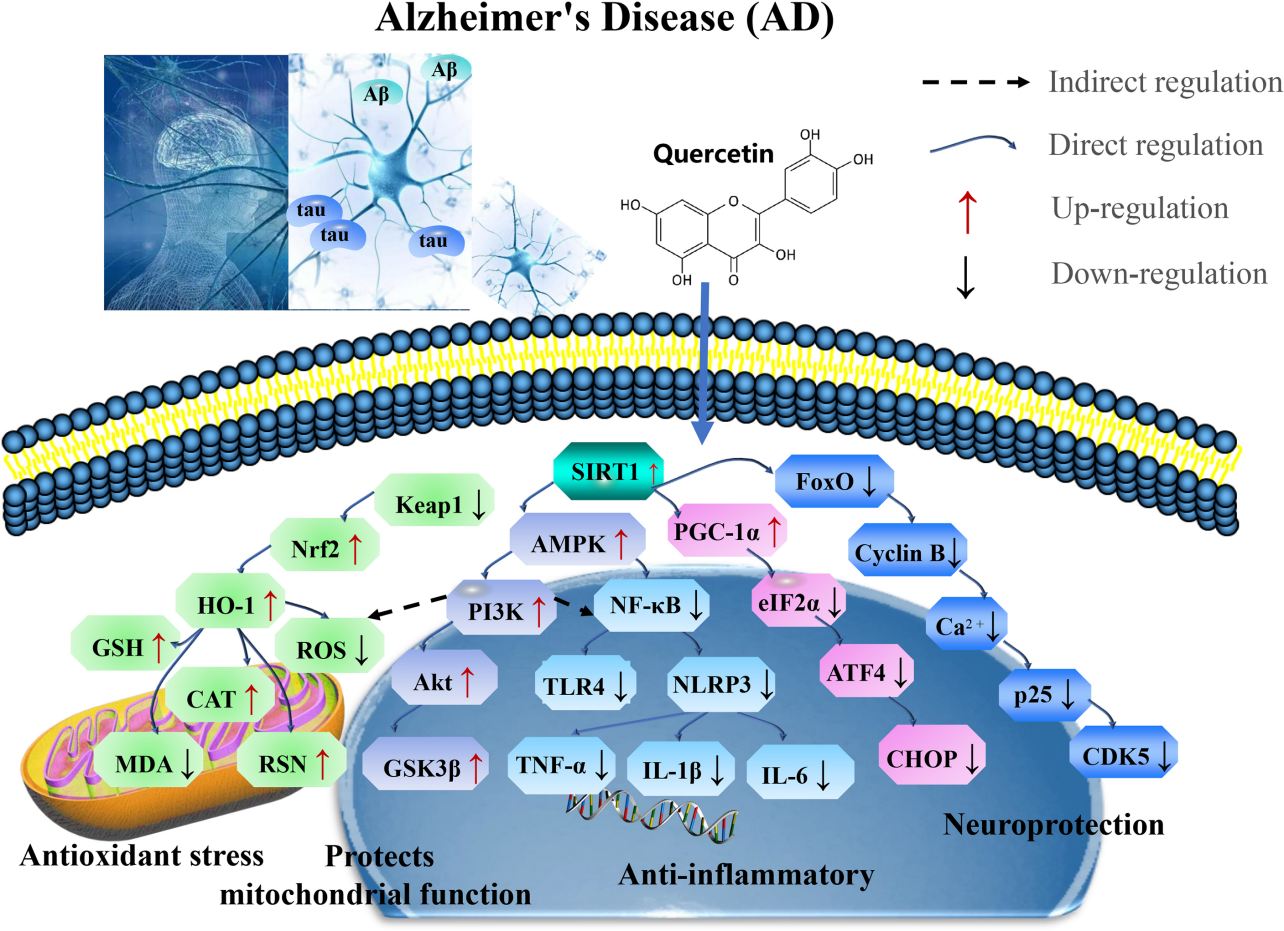
Mechanisms of quercetin on Alzheimer’s disease (AD). Quercetin exert neuroprotective effects against chronic AD by targeting SIRT1 to regulate cellular senescence and aging-related multiple cellular processes, including SIRT1/Keap1/Nrf2/HO-1 and PI3K/Akt/GSK-3β mediated oxidative stress, SIRT1/NF-κB mediated inflammatory response, SIRT1/PGC1α/eIF2α/ATF4/CHOP mediated mitochondrial damage, and SIRT1/FoxO mediated autophagy. CAT, catalase; GSH-Px, glutathione peroxidase; PI3K, phosphoinositide 3-kinase; GSK-3β, glycogen synthase kinase 3beta; iNOS, inducible nitric oxide synthase; TNF, tumor necrosis factor; TLR, toll-like receptors; PGC-1α, proliferator-activated receptor gamma coactivator 1alpha; AMPK, AMP-activated protein kinase; eIF2α, eukaryotic initiation factor 2 alpha; and ATF4, activating transcription factor 4.

In summary, quercetin exerts neuroprotective effects against chronic AD by targeting SIRT1 to regulate cellular senescence and aging-related multiple cellular processes, including SIRT1/Keap1/Nrf2/HO-1 and PI3K/Akt/GSK-3β mediated oxidative stress, SIRT1/NF-κB mediated inflammatory response, SIRT1/PGC1α/eIF2α/ATF4/CHOP mediated mitochondrial damage, and SIRT1/FoxO mediated autophagy (**Figure 2** and **Table 1**).

**Table 1:** Pharmacological functions of quercetin on AD.

*In vitro* and *in vivo* model	Quercetin Dose	Mechanism	Effect factors	References
3xTg-AD mouse	100 mg/kg 2 w	Prevents β-amyloid aggregation	CA1 and tau↓	(47)
APP/PS1 mice	2 mg/g 1 -13 m	Reduce the Aβ and amyloid deposition and astrogliosis	APP, CTFβ, GFAP, Hevin and SPARC↓ p-Smad2 and p-STAT3↑	(55)
AD mice	2 mg/g 16 w	Reduce microglial cell aggregation around amyloid plaques	tau protein↓	(48)
C57BL/6J female mice Atg5KD/SC100/HEK293 cells	50 μM 12 h	Reduce autophagy impairment or ER stress	eIF2α and ATF4↓ APP and IRE1α↑	(61)
APPswe/PS1dE9 transgenic mouse	20, 40 mg/kg 16 w	Activation of AMPK to improve AD	ROA↓ ATP, MMP and AMPK↑	(59)
Drosophila	0.44 g/L 10 d	Decrease extracellular β-amyloidosis, tauopathy, astrogliosis *via* FoxO signaling pathway	APP, cyclin B, BACE1, PS1/2, nicastrin, APH-1, PEN-2 ↓	(64)
Primary Culture of Hippocampal Neurons	20 μM 24 h	Improves mitochondrial function, reduce oxidative stress and apoptosis induction through the Sirt1/PGC-1a axis	caspase-3↓ SIRT1, PGC-1α↑	(60)
PC12 cells	10, 20, 40, 80 μM 24 h	Promote cell proliferation, and antagonize the toxicity of Aβ *via* sirtuin1/Nrf2/HO-1	LDH, AchE ↓ SOD, GSH-P, CAT, T-AOC, sirtuin1, Nrf2 and HO-1↑	(51)
HT22 cells	5, 10 μM 24 h	Enhancement of PI3K/Akt	PSEN1, PSEN2 and APP↓ GST, NQO1, Nrf2, ARE, JNK, AP-1, PI3K, Akt, GSK-3β↑	(52)
HT22 cells	5, 10 μM 24 h	Induce Tau protein activity and blocked the Ca^2+^ -calproteinase-p25-CDK5 signaling pathway	tau protein and Ca^2+^−calpain−p25−CDK5↓	(44)
BV-2 microglia cells	35 μM 24 h	Activate BV-2 microglia at G2/M phase, mitigated inflammatory profile	iNOS, TNF, NF-κB, TLR, NLR, MHC II, CD11B/CR3, CD68↓	(53)

h, hours; d, days; w, weeks; m, months; SOD, superoxide dismutase; CAT, catalase; GSH-Px, glutathione peroxidase; PI3K, phosphoinositide 3-kinase; GSK-3β, glycogen synthase kinase 3beta; iNOS, inducible nitric oxide synthase; TNF, tumor necrosis factor; TLR, toll-like receptors; PGC-1α, proliferator-activated receptor gamma coactivator 1alpha; SPARC, secreted protein acidic and rich in cysteine; ROA, Raman optical activity; MMP, matrix metalloproteinase; AMPK, AMP-activated protein kinase; eIF2α, eukaryotic initiation factor 2 alpha; and ATF4, activating transcription factor 4. ↓ downregulation; ↑ upregulation.

### 2.2 Parkinson’s disease (PD)

Parkinson’s disease (PD) is a common progressive neurodegenerative disease after AD, which affects approximately 1-2% of people at the age ≥ 65 (65). PD is characterized by dopaminergic (DAergic) neuronal deficits and glial dysfunction in the substantia nigra (SN), caused by neuroglial dysfunction and neuroinflammation (66). Studies have shown that α-synuclein (α-SYN) regulates pathological events that cascade the response in PD (67), whereas SIRT1 proteins modulates PD (68).

#### 2.2.1 Regulation of oxidative stress

Oxidative stress is a major inducers of PD pathogenesis. This is because ROS activates α-syn aggregation cascade, together with Lewy bodies, promotes neurodegeneration (69). In a 6-hydroxydopamine (6-OHDA) PD rat model, it was observed that quercetin alleviated the unilateral medial forebrain tract (annigra lesion) or the striatum (part lesion) through the antioxidant, anti-inflammatory, and protective neurotransmitter mechanisms (70–72). The efficacy of quercetin was tested in MitoPark transgenic chronic PD mice model, to ameliorate striatal dopamine depletion and 5-TH neuronal cell loss (73). Other studies have shown that different doses of quercetin reduce oxidative damage by regulating SIRT1/HO-1/Nrf2 pathway, thereby increasing neuronal density (74). Furthermore, Nrf2 modulates ARE/PINK1 expression (75), to restore mitochondrial homeostasis in chronic PD (76).

#### 2.2.2 Regulation of inflammatory response

PD activates microglia to promote inflammatory processes by releasing inflammatory cytokines and chemokines (77). Moreover, degeneration of dopaminergic neurons is induced by the synergy of ROS, neuroinflammation, and loss of other trophic factors (78). In the microglial (N9)-neuronal (PC12) co-culture systems, quercetin downregulates the expression of proinflammatory cytokines in the N9 microglia, and reduce apoptosis in the post-neuronal cells (79). Studies have reported that quercetin alleviates manganese-induced neuroinflammation by inhibiting apoptosis and oxidative stress through the SIRT1/iNOS/NF-κB and HO-1/Nrf2 pathways (80, 81). NLRP3 inflammatory vesicle is one the most characterized inflammatory vesicles, which can be activated by highly diverse stimuli. NLRP3 is activated by procaspase-1, which in turn induces maturation and secretion of inflammatory cytokines, as well as plays an important role in PD (82). Studies have shown that quercetin impedes microglial activation by inhibiting the interaction between the NLRP3 inflammasome and mitochondrial autophagy to reduce neurotoxicity (39). In aged mice, quercetin was reported to attenuate neuroinflammation by modulating the SIRT1/NLRP3 pathway (83).

#### 2.2.3 Regulation of mitochondrial function

Excessive ROS production and accumulation cause mitochondrial dysfunction by α-SYN, which subsequently results in neurodegeneration (80, 84, 85). However, quercetin is significantly induced in MN9D dopaminergic neuronal cells, two major cell survival kinases, and the activation of PD protein kinase D1 (PKD1) and Akt (86). The cAMP response element-binding protein (CREB) and a transcriptional activator of brain-derived neurotrophic factor (BDNF), are involved in microglial activation. Interestingly, quercetin (10 μM) was found to upregulate BDNF gene expression in the dopaminergic neuronal cells through phosphorylating CREB by PKD1 or through stimulating mitochondrial biology in the dopaminergic neuronal cells *via* regulating SIRT1/PGC-1α transcriptional activity through CREB generation. Moreover, mitochondria act as a key regulator for NLRP3 inflammatory vesicles, whereas mitochondrial dysfunction leads to NLRP3 assembly and activation. Mitochondrial dysfunction in the astrocytes amplifies the activity of the NLRP3 inflammasome, as well as promotes IL-1β production.

#### 2.2.4 Regulation of autophagy function

α-SYN regulates autophagosome synthesis, leading to defective autophagy. Quercetin acts as an autophagy enhancer in aged PD rat models and modulate the microenvironment leading to neuronal death (87). PTEN is responsible for inducing putative kinase 1 (PINK1) that contributes to the development of astrocyte and proliferation, as well as was identify as an essential protein for the removal of the damaged mitochondria *via* autophagy (88). PINK1-parkin-mediated dysfunction of the mitochondrial autophagy in the astrocytes can impair the integrity of the mitochondria (89), which leads neuronal dysfunction and degeneration (90). Reports have shown that quercetin activates SIRT1, promotes autophagic PINK1 activation, and reduces cytochrome c release, as well as enable cystatinase activation to maintain mitochondrial integrity, thereby preventing apoptosis (91). Quercetin regulates the protein expression of Bcl2/Bax and the release of cytochrome c, and nuclear translocation of apoptosis-inducing factor (AIF) (92). Quercetin also promotes autophagy and modulates the microenvironment which leads to neuronal death (87).

**Figure 3:**
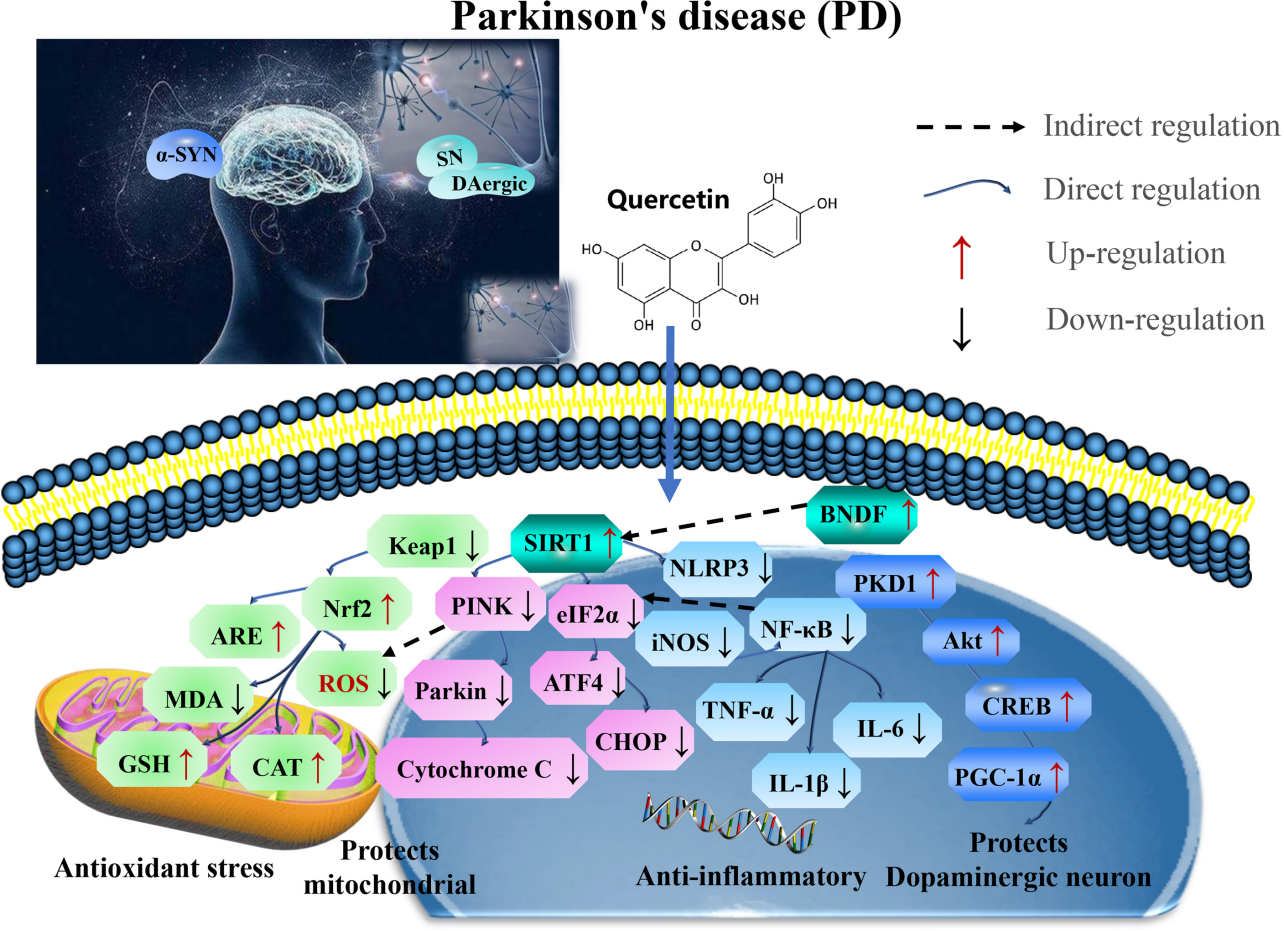
Mechanisms of quercetin on attenuating Parkinson’s disease (PD). Quercetin is a potential therapeutic strategy for PD by targeting SIRT1. Developing therapies have shown that SIRT1/Nrf2/HO-1 mediated oxidative stress, SIRT1/NF-κB/NLRP3 pathway ameliorates neuroinflammation SIRT1-mediated PKD1/CREB phosphorylation and BDNF gene expression, regulates mitochondrial disorders in dopaminergic neurons and SIRT1-PINK1-Parkin mediated mitochondrial autophagy in the astrocytes to maintain mitochondrial function. ROS, reactive oxygen species; 5-HT, 5-hydroxytryptamine; CAT, catalase; GSH-Px, glutathione peroxidase; TNF, tumor necrosis factor; NLRP3, NOD-like receptor protein 3; IL-1β, interleukin-1 β; CREB, cAMP response element binding protein; MDA, malondialdehyde; BDNF, brain-derived neurotrophic factor; and GFAP, glial fibrillary acidic protein.

Therefore, quercetin is a potential therapeutic strategy for alleviating PD by targeting SIRT1. Developing therapies have shown that SIRT1/Nrf2/HO-1 mediated oxidative stress, SIRT1/NF-κB/NLRP3 pathway ameliorates neuroinflammation SIRT1-mediated PKD1/CREB phosphorylation and BDNF gene expression, and regulates mitochondrial disorders in the dopaminergic neurons and SIRT1-PINK1-Parkin mediated mitochondrial autophagy in the astrocytes to maintain mitochondrial function (**Figure 3** and **Table 2**).

**Table 2:** Pharmacological functions of quercetin on PD.

*In vitro* and *vivo* model	Quercetin Dose	Mechanism	Effect factors	References
7-month-old aging mice	35, 70 mg/kg 4 w	Regulates the Sirtuin1/NLRP3 pathway	cleaved caspase 1, IL-1β, IL-18, NLRP3, ASC, MDA and ROS↓ sirtuin1, PSD95, BDNF, NGF and GFAP↑	(83)
parkin+/− x parkin+/− mating mice	5 μM 24 h	Scavenging damaged mitochondria	TBK1‐activated OPTN binding of PINK1‐phosphorylated Ubiquitin.	(91)
6-OHDA rat lesion models	25, 50 mg/kg 6 w	Improves antioxidant and anti-inflammatory potential and restored neurotransmitters	neuroinflammatory (TNF, IL-1 β and IL-6) ↓ LPO, GSH, Nitrite and neurotransmitter (dopamine, norepinephrine, serotonin, GABA, glutamate)↑	(72)
rats to cadmium	25 mg/kg 28 d	Modulates mitochondrial integrity and MAP Kinase signaling	ROS, MAPK, c-Jun N, p38 and ERK↓ PKCβ1, ChAT and AChE↑	(84)
Rotenone- and Iron Supplement–Induced Parkinson disease in Experimental Rats	25, 50 mg/kg 28 d	Neuroprotective effect *via* antioxidant, anti-inflammatory	TNF, IL-1β and IL-6↑ LPO, GSH, mitochondrial complexes I and IV↑	(80)
rotenone rat model of PD	2 ml/kg 4 w	Augmentes autophagy, ameliorated ER stress-induced apoptosis with attenuated oxidative stress	C/EBP homologous protein (CHOP), Beclin-1, and dopamine↑	(87)
Transient transfections of MN9D cells 12-week-old MitoPark mice	10, 30 µM 24 h 25 mg/kg 6 w	Up-regulates mitochondrial complex-I activity to repair mitochondrial	Complex-I↓ Cu/Zn- and Mn-SOD, catalase, GSH and GSSG↑	(73)
Adult male Wistar rats	100, 200, 300 mg/kg 6 d	Decrease oxidative damage resulting in increased neuron density.	AChE, MDA ↓ GPx, L-dopa, vitamin C, SOD and CAT↑	(74)
PD mouse authenticated mouse microglia BV2 cells	30 μM 1 h	Reduce mtROS accumulation and alleviated NLRP3 inflammasome activation.	ROS, iNOS, IL-1β, IL-6, TNF, NLRP3 and caspase-1↓ Nrf2↑	(39)
mouse dopaminergic MN9D cells	10 µM 1 h	Activate PKD1-Akt cell survival signaling axis	PKD1, Akt, PGC-1α, CREB ​​and BDNF↑	(86)
rat pheochromocytoma (PC-12) cells	10, 50,100 µM 24 h	Neuroprotective effects as effective antioxidants	CuZn-SOD, Mn-SOD, CAT, GSH and GSH-Px↑	(71)
Microglial (N9)-Neuronal (PC12) Coculture System	0.1 μ M 3 h	Rescue neuronal PC12 cells from glial-evoked apoptosis	IL-6, IL-1β, TNF or iNOS ↓	(79)
SK-N-MC human neuroblastoma cell line and Sprague-Dawley (SD) male rat brain	10, 20 µg/mL 16 w	Alleviate oxidative stress through regulation of apoptosis, iNOS/NF-κB and HO-1/Nrf2 pathways	ROS, TNF-, TNF, IL-1β, IL-6, COX2, iNOS, Bax, Cytochrome, Caspase-3 and PARP-1↓ SOD, Bcl-2↑	(79)
Rat PC12 cells	0.1 μM 3 h	Preventive neurodegenerative diseases caused by oxidative stress and apoptosis.	LDH, Bax, caspase-3, Cytochrome c and AIF↓ Bcl-2↑	(92)
6-OHDA rat lesion models SH-SY5Y neuroblastoma cells	10–100 μM 50–200 mg/kg	Reliable neuroprotective effects	ROS↓ 5-HT, 5-HIAA↑	(70)

h, hours; d, days; w, weeks; ROS, reactive oxygen species; 5-HT, 5-hydroxytryptamine; SOD, superoxide dismutase; CAT, catalase; GSH-Px, glutathione peroxidase; TNF, tumor necrosis factor; NLRP3, NOD-like receptor protein 3; IL-1β, interleukin-1 β; CREB, cAMP response element binding protein; MDA, malondialdehyde; BDNF, brain-derived neurotrophic factor; GFAP, glial fibrillary acidic protein; LDH, lactate dehydrogenase; and AIF, apoptosis-inducing factor. ↓ downregulation; ↑ upregulation.

### 2.3 Effects of quercetin on Huntington’s disease (HD)

Huntington’s disease (HD) is a neurodegenerative disorder characterized by a progressive loss of dopaminergic neurons in the substantia nigra, as well as progressive motor dysfunction, chorea, dystonia, mood disturbances, memory, and weight loss. Symptoms of HD in humans usually appear between the ages 30-50 years and increase in severity with chronological age (93). Current disease palliative therapies for HD focus on reducing the levels of mutant huntingtin (mHTT) in brain cells (94). The overexpression of SIRT1 was reported to increase the survival in R6/2 HD mice and improve neuropathology and reduced mHTT aggregation in R6/2 models (95).

#### 2.3.1 Regulation of mitochondrial function

In 3-nitropropionic acid (3-NP)-induced HD rat models, quercetin maintain mitochondrial function by attenuating oxidative stress and neurobehavioral disorder (96). In addition, dysfunction of mitochondrial metabolism of mHTT play major role in the pathogenesis of HD which is primarily associated with impair respiratory chain function, thereby increasing ROS production and subsequently results in cell death. Furthermore, the ubiquitin-proteasome system (UPS) or autophagy reduce the formation of toxic mHTT aggregates (97). A study showed that inducement of mHTT could decrease the proteasomal activity of UPS which could be reverse by the quercetin (20 µM), thereby attenuating mitochondrial membrane potential damage (98).

#### 2.3.2 Regulation of inflammatory response

**Figure 4:**
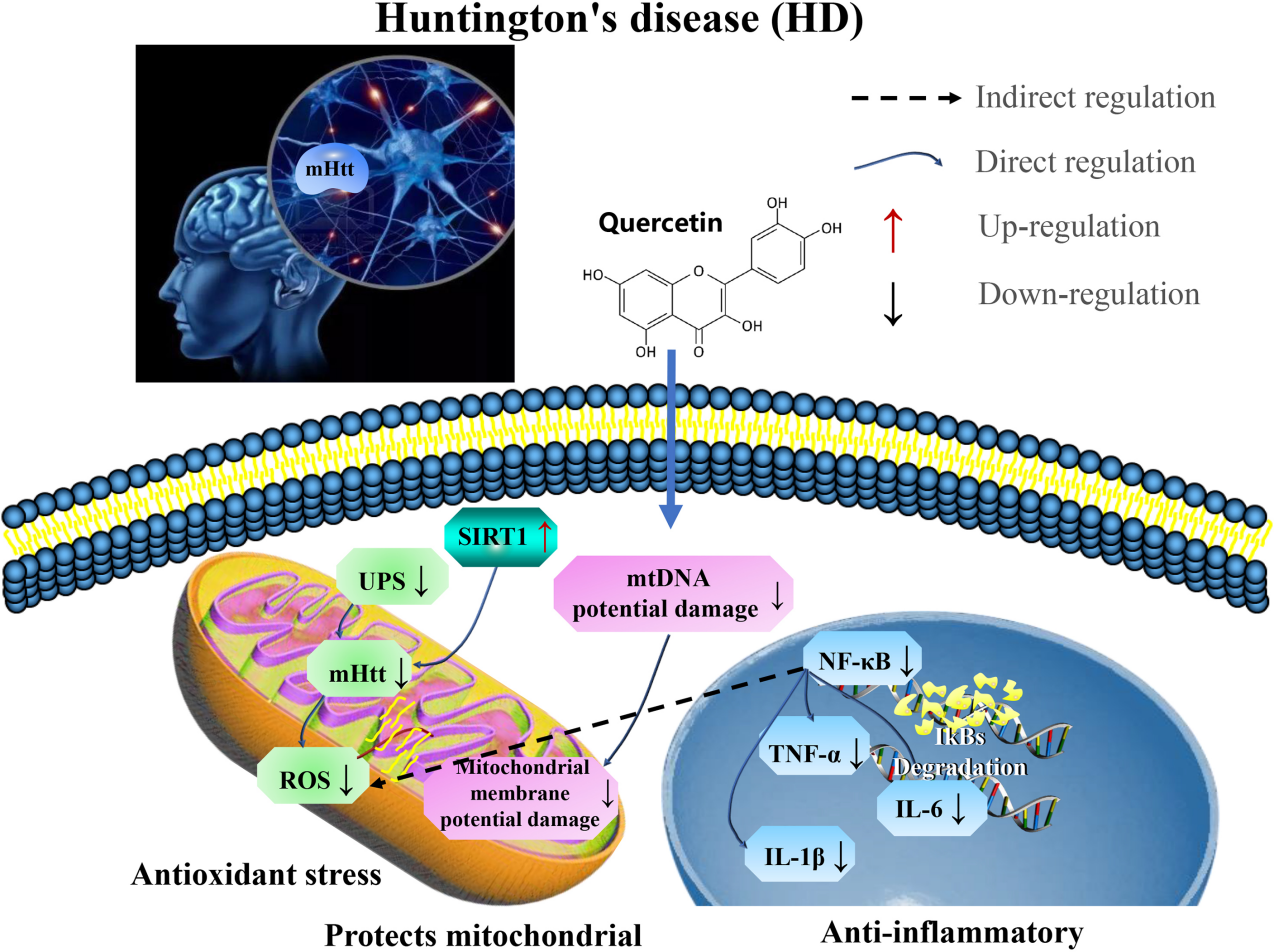
Mechanisms of quercetin in attenuating Huntington’s disease (HD). Quercetin plays neuroprotective role in HD by targeting SIRT1 to relieve aggregation of mHTT in patients, restore mitochondrial function, and reduce inflammation. UPS, ubiquitin-proteasome system; mHtt, Huntington’s protein; ROS, reactive oxygen species; TNF-α, tumor necrosis factor-α; and IL-1β, interleukin-1 β.

It was established that quercetin could alleviate HD in rat model by regulating inflammatory changes (IL-1β, IL-6, and TNF) associated with growth factors released from astrocytes by the reduction of microglial proliferation, as well as increasing astrocyte numbers (99). In addition, quercetin reduce anxiety and depression in patients with 3-NP-induced chronic HD (100). This indicate that quercetin plays neuroprotective role in HD by targeting SIRT1 to relieve mHTT aggregation in patients, and restore mitochondrial function and reduce inflammation (**Figure 4** and **Table 3**).

**Table 3:** Pharmacological functions of quercetin on HD.

*In vitro* and *vivo* model	Quercetin Dose	Mechanism	Effect factors	References
150Q mutated huntingtin-expressing cells	20 µM 4 d	Improves the activity of the ubiquitin proteasomal system and upregulated UPS	UPS, mHtt↓	(98)
3‐Nitropropionic Acid‐Induced Rat Model of Huntington’s Disease	20 mg/kg 4 d	Improves the motor coordination, locomotor functions, and anxiety	Cd11B and glial fibrillary acidic↓	(99)
3-NP-induced male Wistar rats	50mg/kg 14 d	Alleviates anxiety and depression	body weight and locomotion count↑	(100)
3-NP-induced female Wistar rats	25 mg/kg 21 d	Against mitochondrial oxidative stress, mitochondrial dysfunctions and neurobehavioral deficits.	MDA, ATP levels and ATP/ADP↓ SOD, CAT↑	(96)

d, days; UPS, ubiquitin-proteasome system; mHTT, mutant huntingtin; MDA, malondialdehyde; SOD, superoxide dismutase; CAT, catalase; 3-NP, 3-nitropropionic acid. ↓ downregulation; ↑ upregulation.

### 2.4 Effects of quercetin on depression

Major depressive disorder may be associated with volumetric indications of accelerated brain aging (101). Disorders of depression and anxiety are common mental ailments that imposes a significant global health challenge. However, current conventional antidepressants have limited efficacy, significant side effects and expensive. The structural and functional integrity of the hippocampus is critical for cognitive functions, as well as producing antidepressant effects in response to adverse factors (environmental changes, stress) (102, 103). Moreover, reports have indicated that SIRT1 inhibitors reverse sleep deprivation (SD)-induced depressive and anxiety-like behaviors and hippocampal neuroinflammation (104). The role and possible mechanisms of SIRT1 have revealed novel therapeutic strategies for clinical treatment of depression (105, 106). SIRT1 is required for normal neuronal excitability and regulates depression-related behaviors in a sex-specific manner (107). Activation of the estrogen receptor alpha (ERα)/SIRT1/NF-κB pathway was involved in LPS-induced depression in aged female mice (108).

Fork head box transcription factor G1 (FOXG1) play neuroprotective roles by regulating adult hippocampal neurogenesis (AHN). Quercetin promotes AHN *via* FoxG1/CREB/BDNF signaling pathway to improve chronic unpredictable mild stress (CUMS)-induced depression-like behaviors (109). In addition, it has been shown that microglial inhibitory pathways exert neuroprotective effects, and astrocyte activation, whereas quercetin significantly reduce the frequency of spontaneous excitatory postsynaptic currents (sEPSCs) and spontaneous inhibitory postsynaptic currents (sIPSCs) to antidepressants (110). Astrocytes secrete neurotrophic factors such as GDNF and BDNF, of which BDNF combined with exercise training attenuated 1,2-dimethylhydrazine-induced depression in rats with colorectal cancer by modulating the BDNF/tyrosine receptor kinase A (TrKβ)/β-catenin axis in the prefrontal cortex significantly reduced tumorigenesis and improve depression-like behavior (111), as well as attenuates LPS-induced depression-like behavior in rats by modulating the BDNF-related Copine 6 and TREM1/2 imbalance in the hippocampus and PFC (112), whereas reports showed that quercetin exert antidepressant and cardioprotective effects (113).

Quercetin reduce doxorubicin-induced anxiety by enhancing immune function and reducing oxidative stress in the brain (114). Alteration in the metabolism of monoamine oxidase (MAO) is associated with aging (115), which catalyzes monoamine-containing neurotransmitters such as serotonin 5-hydroxytryptamine (5-HT) (116). Quercetin possess antidepressant-like effects through its antioxidant, anti-inflammatory activities, and also reduce excitotoxicity, as well as increase 5-HT levels (117). Quercetin significantly reduced MAOs activity and increase the activity of antioxidant enzymes (Cu-Zn, SOD, CAT, and GSH-Px) in depressed rats (118). Some studies in olfactory bulbectomy (OB), the surgical removal of the olfactory bulbs, cause specific set of behavioral changes in social behavior, cognitive function, and activity; whereas quercetin shows an antidepressant-like and antioxidant effects in bulbectomized mice model *via* the glutamatergic and oxidonitrergic pathways (119), and mediate neuroinflammatory-apoptotic cascade response (120).

**Figure 5:**
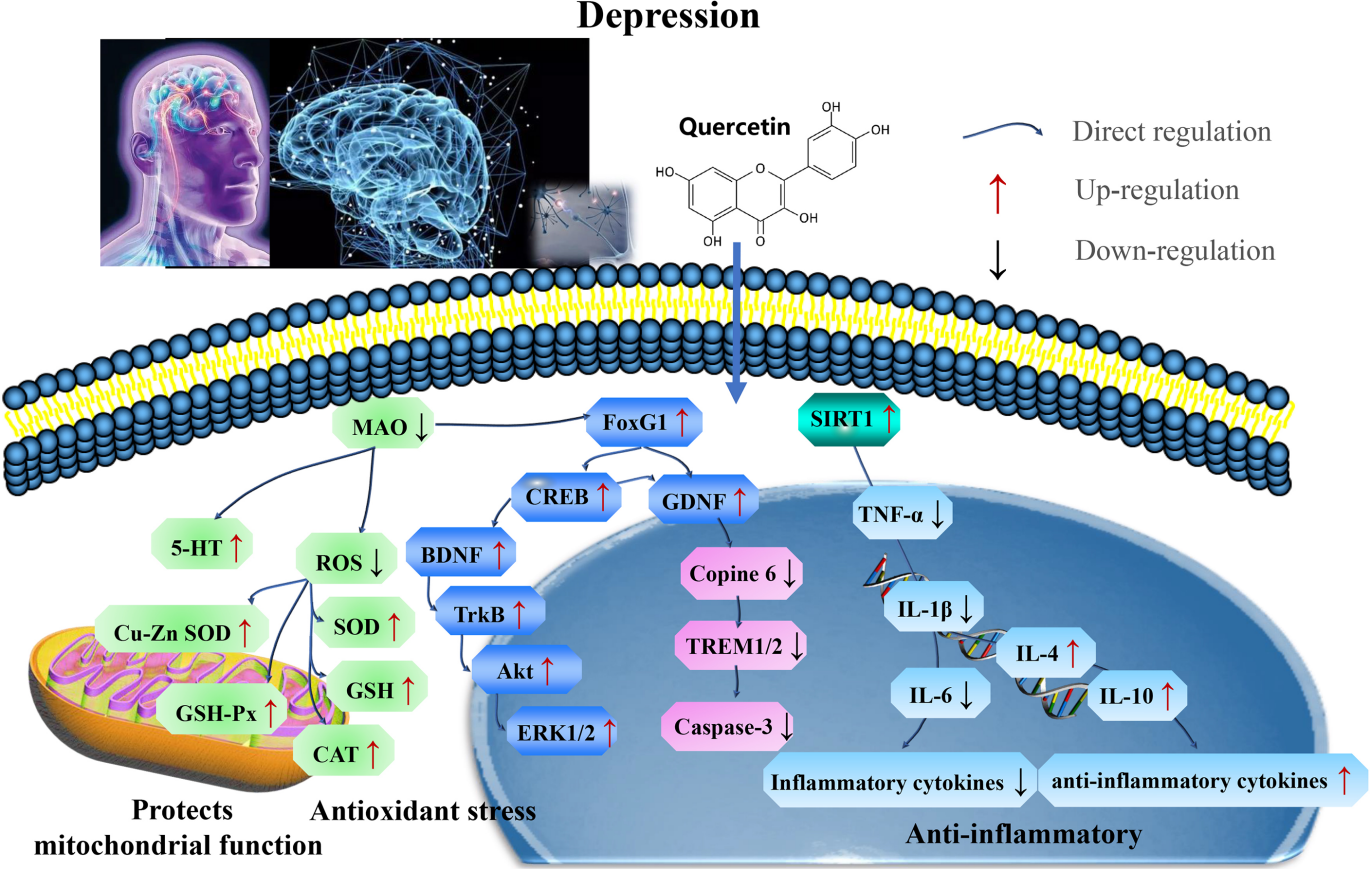
Mechanisms of quercetin on Depression. Quercetin act as an antidepressant by targeting SIRT1 to reverse depressive and anxiety-like behaviors and hippocampal neuroinflammation. The FoxG1/CREB/BDNF/Trkβ-catenin axis clarifies these mechanisms. FOXG1, Forkhead box transcription factor G1; CREB, cAMP response element binding protein; TrKβ, tyrosine receptor kinase A. SOD, superoxide dismutase; CAT, catalase; GSH-Px, glutathione peroxidase; TNF, tumor necrosis factor; IL-1β, interleukin-1 β; MDA, malondialdehyde; and BDNF, brain-derived neurotrophic factor.

Furthermore, quercetin acts as an antidepressant by targeting SIRT1 to reverse depressive and anxiety-like behaviors and hippocampal neuroinflammation. The FoxG1/CREB/BDNF/Trkβ-catenin axis clarify the underlying mechanisms (**Figure 5** and **Table 4**).

**Table 4:** Pharmacological function of quercetin on depression.

*In vitro* and *in vivo* model	Quercetin Dose	Mechanism	Effect factors	References
Male ICR mice	15, 35mg/kg 4 w	Promotes adult hippocampal neurogenesis *via* FoxG1/CREB/BDNF signaling pathway	FoxG1, p-CREB and BDNF↑	(109)
Rats	10, 50mg/kg 8 w	Reduces oxidative stress, inhibited inflammation, and regulated a variety of neurotransmitter systems.	MAO, IL-1β and TNF-α↓ Cu-Zn SOD, GSH-Px, CAT and GSH↑	(118)
mice	2, 0.5g/kg 8 w	Antidepressant and cardioprotective effects *via* BDNF-AKT/ERK1/2 signaling	BDNF-TrkB-AKT/ERK1/2↑	(113)
mice	2, 0.5 g/kg 6 w	Improves mice behavioral performance post CSDS. Decreases sEPSCs and sIPSCs	sEPSCs and sIPSCs↑	(110)
Mice	25 mg/kg 6 w	Antioxidant, anti-inflammatory activities, reduced excitotoxicity and augmented 5 HT levels.	TNF and IL-6↓ SOD, GSH, Catalase and 5 HT↑	(117)
depression in rats	40 mg/kg 8 w	Alleviates LPS-induced depression-like behaviors *via* regulating the BDN/Copine 6 and TREM1/2	TNF, IL-6, caspase-3↓	(112)
male wistar rat	60 mg/kg 24 h	Against chemotherapy-related complications	MDA, TNF, ROS/RNS↓ GSH	(114)
OBX-induced depression in male Wistar rats	40, 80 mg/kg 14 d	Suppression of oxidative–nitrosative stress-mediated neuroinflammation-apoptotic cascade	MDA↓ GSH, SOD↑	(120)
Olfactory bulbectomy (OB)	25mg/kg 14 d	Antioxidant effects contribute to its anti-depressive potential	LOOH↓ GSH, SOD↑	(119)

h, hours; d, days; w, weeks; FOXG1, Forkhead box transcription factor G1; CREB, cAMP response element binding protein; TrKβ, tyrosine receptor kinase A; SOD, superoxide dismutase; CAT, catalase; GSH-Px, glutathione peroxidase; TNF, tumor necrosis factor; IL-1β, interleukin-1 β; MDA, malondialdehyde; and BDNF, brain-derived neurotrophic factor. ↓ downregulation; ↑ upregulation.

## 3 Effects of quercetin on osteoporosis

Osteoporosis is also a major aging-related disease (121). The rate of reduction of osteogenic differentiation and bone formation is the major cause of aging-related osteoporosis (122). Bone can act indirectly on the brainstem, midbrain and hippocampus, thereby affect the synthesis of multiple neurotransmitters, resulting in neurodegenerative diseases (123), suggesting that improving the conditions affecting neurodegenerative process may be a novel target for the treatment of bone injury. Osteoporosis is characterized by a reduction in the bone mass and bone mineral loss, and deterioration of bone microarchitecture, with a greater impact on postmenopausal women. In the United States, approximately 53 million people are at risk of bone mineral loss, however, this number will gradually increase resulting in an economic loss of an annual cost of $25.3 billion (124).

Quercetin is a potential drug for the clinical treatment of osteoporosis and has a significant effect on the structure and conformation of bone morphogenetic protein-2 (BMP-2) *via* upregulation of bone mineralization which promotes differentiation of bone marrow mesenchymal stem cells (BMSCs), as well as osteoblast-specific genes such as osterix (OSX), dwarf-related transcription factor 2 (Runx2), alkaline phosphatase (ALP), osteocalcin (OCN), and serum c-terminal type I collagen cross-linked telopeptides at the mRNA and protein expression levels (40, 125). Quercetin promotes osteogenic differentiation and inhibits lipogenic differentiation of mouse bone marrow mesenchymal stem cells (mBMSCs), enhancing AMPK protein phosphorylation and upregulating SIRT1 expression to exert antioxidant effects (126). Thereby promoting bone marrow mesenchymal stem cell proliferation and osteogenic differentiation. Rodent oophorectomy is extensively studied for to determine a reduction in bone mass and an increase in bone turnover in cancellous bone, which is similar to osteoporosis in postmenopausal women (127).

The activation of nicotine and muscarinic receptors (nAchR) in an osteoclast inhibits the receptor activator of nuclear factor-kappa B ligand (RANKL)-dependent osteoclast development, suggesting that it is closely related to sympathetic nerve activity (128). Quercetin has been shown to inhibit RANKL-mediated osteoclastogenesis, osteoblast apoptosis, and inflammatory response, nuclear factor κB (NF-κB) and activator protein 1 (AP-1) are transcription factors that regulates osteoclast differentiation, and signaling pathways associated with β-catenin degradation (129). Quercetin is inhibits osteoclast differentiation *in vitro* (130). The abundance of phosphorylated p38 MAPK and phosphorylated extracellular signal-regulated kinase (ERK) could be reverse after quercetin treatment, and trigger significant restoration of the Wnt/β-catenin pathway by enhancing the expression of Wnt3, β-catenin, Bax and cytochrome c expression and decrease the expression of Bcl-2, Bcl-xL and caspase-3 (131), regulating autophagy and apoptosis to prevent osteoporosis (132).

**Figure 6:**
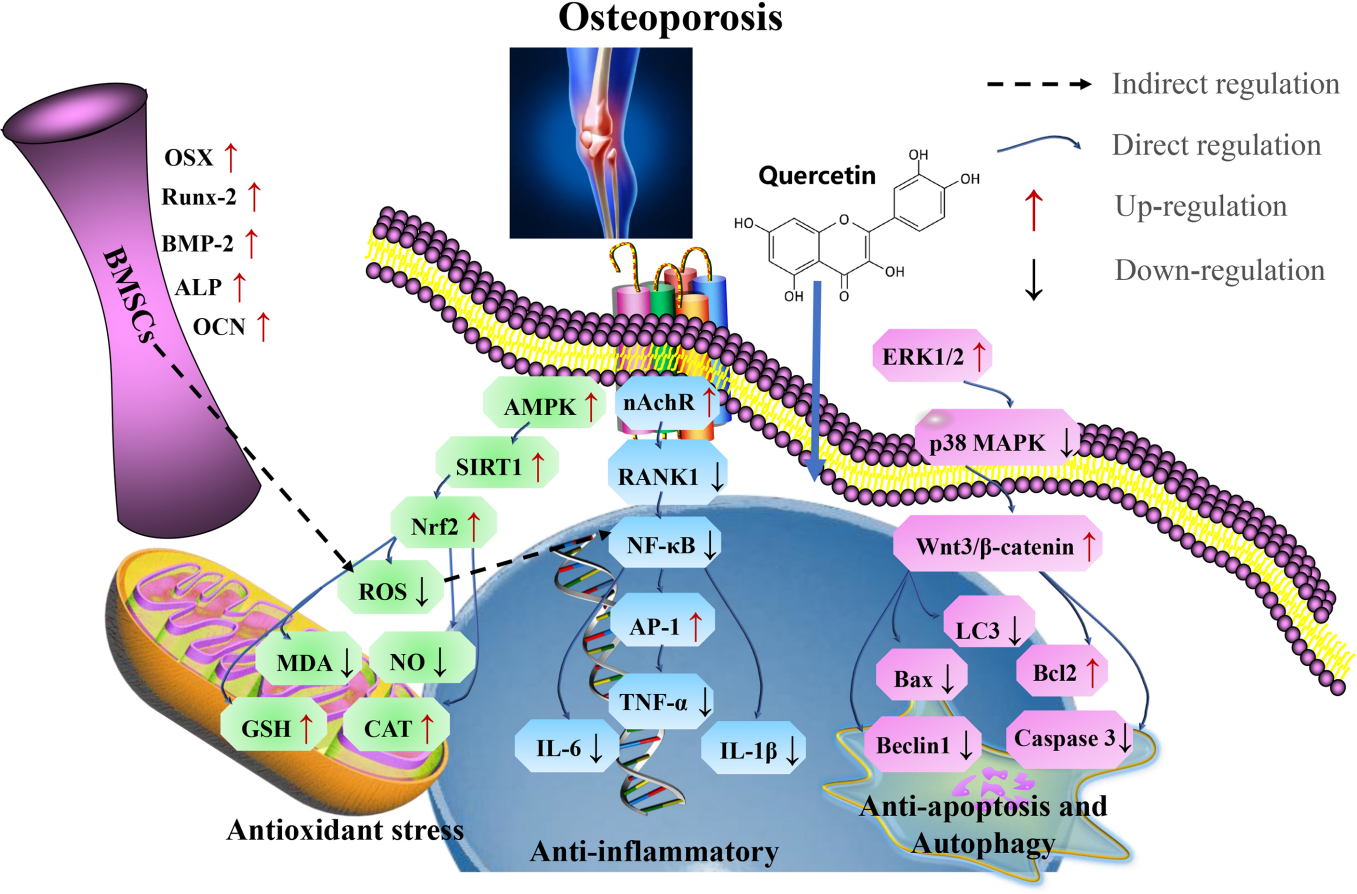
Mechanism of quercetin on osteoporosis. Quercetin as a therapeutic strategy for the treatment of aging-related osteoporosis by targeting SIRT1, *via* antioxidant pathways, thereby inhibits osteoblast apoptosis, autophagy, and inflammatory responses. Runx2, related transcription factor 2; OSX, Osterix; OCN, osteocalcin; Cx43, connexin 43; RANKL, receptor activator of nuclear factor-kappa B ligand; TNF, tumor necrosis factor; IL-6, interleukin-6; IFN-γ, interferon γ; SOD, superoxide dismutase; CAT, catalase; GSH, glutathione; ALP, alkaline phosphatase; LC3, microtubule-associated protein light chain 3; OPG, osteoprotegerin; CTX-1, C-terminal telopeptide of type I collagen; P1NP, N-terminal propeptide of type I procollagen; TRAP, Tartrate-resistant acid phosphatase; Runx2, related transcription factor 2.

In summary, quercetin acts as a therapeutic measure for treating aging-related osteoporosis by targeting SIRT1, *via* antioxidants pathways, thereby inhibits osteoblast apoptosis, autophagy, and inflammatory responses (**Figure 6** and **Table 5**).

**Table 5:** Pharmacological functions of quercetin on osteoporosis.

*In vitro* and *in vivo* model	Quercetin Dose	Mechanism	Effect factors	References
mice were used for isolating the primary BMSCs	2, 5 μM 24 h	Promotes antioxidant *via* activation of the AMPK/SIRT1	CTX↓ ALP and OC↑	(126)
rat bone cells	15, 7.5 mg/kg 10 w	Prevents osteoporosis by regulating the total number of bone cells, maybe through regulating autophagy and apoptosis.	LC3, beclin1, and caspase 3↓ Bcl2↑	(132)
human osteoblast cell line (MG-63)	25-200 ppm 2 d	Activates osteoprotegerin (OPG) and inhibited RANKL expression	OC and RANKL↓ ALP, OPG and collagen ↑	(133)
MC3T3-E1 Cells	10, 25, 50 µM 2 h	Inhibition Apoptosis *via* the MAPK and Wnt/β-Catenin signaling pathways	OSX, Runx2, ALP and OCN↑ caspase-3, Bax, cytochrome c, phosphorylated MAPKs and Wnt/β-catenin↓ Bcl-2, Bcl-XL ↑	(131)
RAW 264.7 cells	1-10 μM 24 h	Decrease osteoclastic differentiation induced by RANKL	NF-κB, AP-1↓	(129)
female SD rats	50 mg/kg 8 w	Promotes BMSC proliferation and osteogenic differentiation against TNF-α-induced impairments	TNF-α, NF-κB and β-catenin↓ Runx2 and Osterix↑	(134)
female SD rats	50, 100, 200 mg/kg 60 d	Downregulates MAPK signaling pathways and preventes the ovariectomy-induced deterioration of bone mineral density (BMD)	CTX-1, TRAP↓ Ca, P, ALP, and P1NP ↑	(135)

h, hours; d, days; w, weeks; Runx2, related transcription factor 2; OSX, Osterix; OCN, osteocalcin; Cx43, connexin 43; RANKL, receptor activator of nuclear factor-kappa B ligand; TNF, tumor necrosis factor; IL-6, interleukin-6; IFN-γ, interferon γ; SOD, superoxide dismutase; CAT, catalase; GSH, glutathione; ALP, alkaline phosphatase; LC3, microtubule-associated protein light chain 3; OPG, osteoprotegerin; CTX-1, C-terminal telopeptide of type I collagen; P1NP, N-terminal propeptide of type I procollagen; TRAP, Tartrate-resistant acid phosphatase; and Runx2, related transcription factor 2. ↓ downregulation; ↑ upregulation.

## 4 Effect of quercetin on aging-related cardiovascular disease

### 4.1 Myocardial ischemia (M/I) and reperfusion (MI/R)

Myocardial ischemia is an aging-related cardiovascular disease and causes sudden death worldwide (136). Myocardial ischemia (M/I) infarction is caused by an inadequate supply of oxygen to the heart, leading to apoptosis (137). However, cardiomyocytes damage is caused by calcium overload, large amounts of free radical production, and infiltration of inflammatory cells cause reperfusion. Mitochondrial dysfunction is considered an important marker of neuronal death during cerebral MI/R (7).

The cardioprotective effect of quercetin is associated with attenuating oxidative stress induced by aging (138). In a mice model, quercetin was reported to attenuate cardiac damage induced by a high-fat diet (HFD) by restoring myocardial microcirculation, reducing infarct size and improving left ventricular function, myofibrillar and mitochondrial structure (139). Quercetin also improve the tricarboxylic acid cycle and respiratory chain-related enzyme activity in rats with myocardial infarction, as well as decrease the expression of biomarkers of myocardial induced oxidative stress in rats with myocardial infarction (140). In addition, quercetin protects AC16 cells from HG-induced oxidative stress by elevating p-SIRT1, endothelial NOS, and decreasing iNOS (141) through the PI3K/Akt/Nrf2 signaling pathway (142).

Pretreatment with quercetin significantly inhibit inflammatory cascade responses through the downregulation of HMGB1-TLR4-NF-κB signaling pathway (143). Quercetin protects against myocardial apoptosis *in vivo via* phosphorylation of JNK and p38, increasing Bcl-2 expression and directly or indirectly inhibiting the activation of Bax and caspase 3 (144, 145), as well as ameliorates MI/R-induced apoptosis through SIRT1/PGC-1α signaling (146).

Evidence shows that sympathetic excitatory reflexes exacerbate myocardial injury (147). In a neuropathic pain model a central mechanism through the inhibition of mitochondrial permeability transition pore (mPTP) opening (148) or involving the activation of paraventricular thalamic (PVA) neurons (149) induce cardioprotective effects.

**Figure 7:**
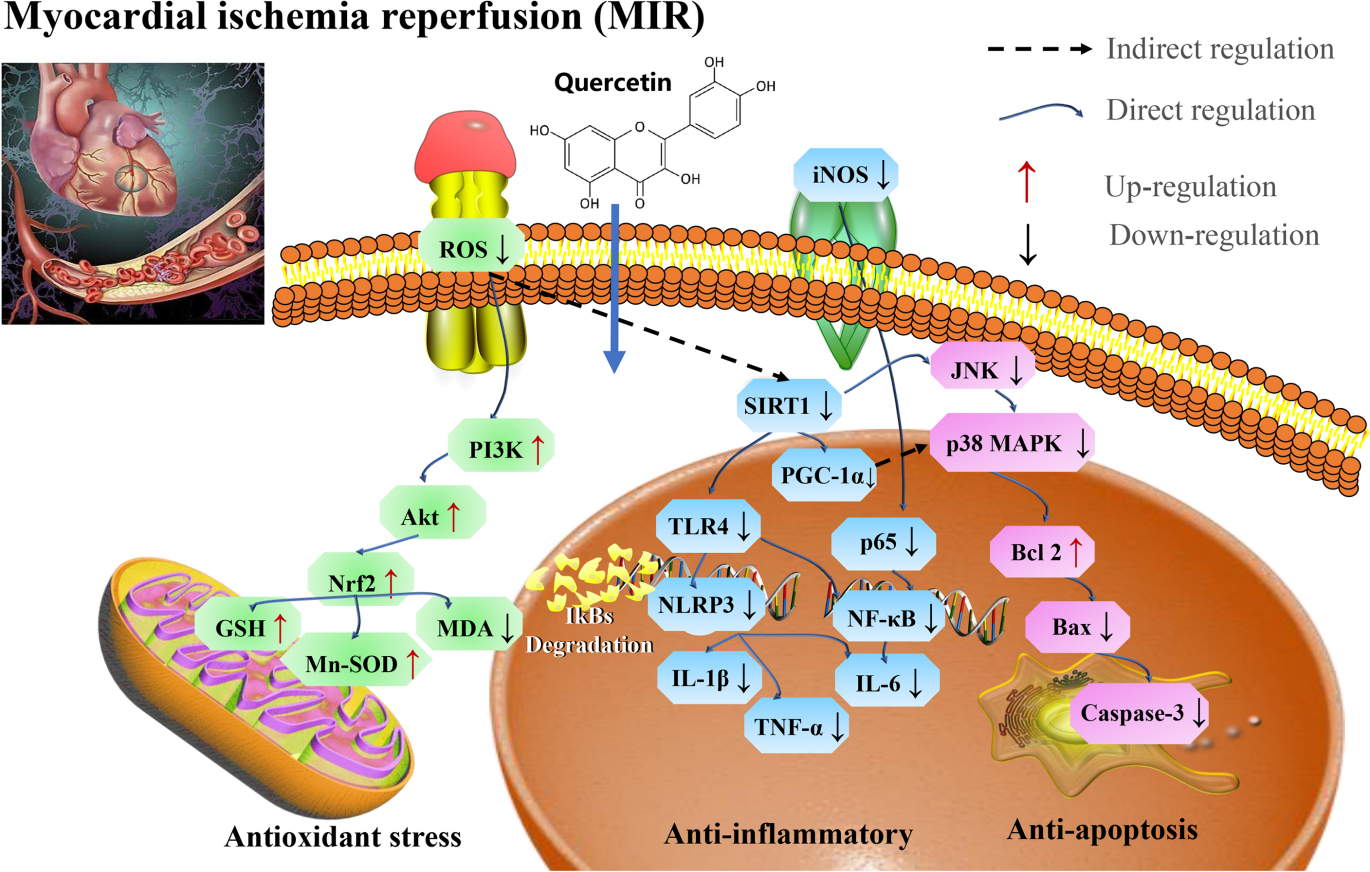
Mechanisms of quercetin on Myocardial ischemia (M/I) and reperfusion (MI/R). Quercetin is potential therapeutic drug that play roles in reducing MI/R injury *via* SIRT1/PI3K/Akt/Nrf2 mediated oxidative stress, SIRT1/PGC1α and HMGB1/TLR4/NF-κB mediated inflammatory response, and SIRT1/p38 MAPK mediated apoptosis pathways. MDA, malondialdehyde; SOD, superoxide dismutase; TNF-α, tumor necrosis factor-α; IL-1β, interleukin-1 β; iNOS, inducible nitric oxide synthase; GSH-Px, glutathione peroxidase; TLR4, toll-like receptor 4; and PGC-1α, proliferator-activated receptor gamma coactivator, 1alpha.

Therefore, quercetin could be used as a potential therapeutic drug for reducing MI/R injury *via* SIRT1/PI3K/Akt/Nrf2 mediated oxidative stress, SIRT1/PGC1α and HMGB1/TLR4/NF-κB mediated inflammatory response, and SIRT1/p38 MAPK mediated apoptosis pathways (**Figure 7** and **Table 6**).

**Table 6:** Pharmacological function of quercetin on myocardial ischemia and reperfusion.

*In vitro* and *in vivo* model	Quercetin Dose	Mechanism	Effect factors	References
C57BL/6J mice	50 mg/kg 10 w	Induces angiogenesis and decreased myocardial oxidative stress	HOMA-IR↓	(138)
rats	50 mg/kg 7 d	Upregulation of antioxidants and activation of STAT3.	NF-κB, p62, TNF-α, IL-6 and MDA ↓ SOD, GSH‐Px and CAT↑	(150)
a rat with AMI	80 mg/kg 2 w	Ameliorates anti-inflammation and anti-apoptosis factor and regulated TLR4-NF-κB signal pathway	MDA, IL-6, TNF-α, caspase-3 activity and Bax/Bcl-2↓ SOD ↑	(141)
acute myocardial infarction (AMI) rats	100, 400 mg/kg 1 w	Anti-inflammatory and antioxidant myocardial protective mechanisms	MDA, TNF-α, and IL-1β↓ SOD and CAT↑	(151)
SD rats	250 mg/kg 1 w	Decrease oxidative stress, repress inflammatory cascade, inhibits apoptosis *in vivo* and PI3K/Akt pathway involved in the anti-apoptotic effect	MDA, TNF-α, CRP and IL-1β↓ SOD, CAT and GSH-PX↑	(145)
AC16 cells	1, 5, 10, or 20 μM 48 h	Against high glucose-induced injury, oxidative stress, and apoptosis by activation of PI3K/Akt/Nrf2 pathway	ROS, MDA and cytochrome c ↓ SOD, GSH‐Px and CAT↑	(142)
cardiomyocyte	25, 50, 100 mg/kg 2 w	Reduce apoptosis *via* SIRT1/PGC-1α signaling	SIRT1, PGC-1α, Bcl-2↑ Bax↓	(146)
Adult SD rats H9C2 rat cardiomyocyte cells	50 mg/kg 5 d 40μM 24 h	Downregulation of the HMGB1-TLR4-NF-κB signaling pathway	IL-6, IL-1β, TNF-α, TLR4, HMGB1 and p-NF-κB ↓	(143)
Male C57/BL6-mice Myocardial H9C2 cells	40 μM 24 h	Improves cardiac function, diminishes myocardial injury and reduce the infarct size *via* suppressing the NF-κB	MDA, PPARγ, NF-κB and PI3K/Akt ↓ SOD, GSH-PX and Bcl-2↑	(152)
H9c2 cardiomyocyte cells	10, 20, 40, 80, 160 μM 4 h	Inhibition of JNK and p38	Bcl-2↑ Bax and caspase-3↓	(144)

h, hours; d, days; w, weeks; HOMA-IR, homeostasis model assessment of insulin resistance; MDA, malondialdehyde; SOD, superoxide dismutase; CAT, catalase; TNF-α, tumor necrosis factor-α; IL-1β, interleukin-1 β; iNOS, inducible nitric oxide synthase; GSH-Px, glutathione peroxidase; PPARγ, peroxisome proliferator-activated receptor gamma; TLR4, toll-like receptor 4; HMGB1, high mobility group box-1; PGC-1α, proliferator-activated receptor gamma coactivator, 1alpha; ↓, downregulation; ↑, upregulation.

### 4.2 Atherosclerosis (AS)

Atherosclerosis promotes mortality and morbidity in aging-related cardiovascular diseased models (8, 153). Endothelial dysfunction is an important process involved in atherosclerosis (154). A solute exchange between the blood and nerve tissues have a direct contact and are intact by the blood-nerve barrier (BNB), consisting of the endothelium surrounding peripheral nerve substructures (155, 156). Oxidized low-density lipoprotein (ox-LDL)-induced oxidative damage to endothelial cells cause atherosclerosis. Thus, oxLDL-induces the formation of RAW264.7 macrophage-derived foam cells that exacerbate cellular lipid accumulation, and elevate the levels of ROS that lead to oxidation of LDL particles to produce ox-LDL, whereas quercetin inhibits cholesterol accumulation-induced apoptosis of macrophages, thereby reducing atherosclerosis (157, 158). In addition, quercetin enhances cellular antioxidant function through the Nrf2 pathway (154). Further studies have showed that quercetin inhibits ox-LDL induced oxidative damage in AS *via* activating SIRT1 and modulating the AMPK/NADPH/AKT signaling pathway (159), as well as inhibits inflammatory/oxidative stress responses in AS *via* AMPK/SIRT1/NF-κB pathway (160), and attenuated AS by increasing the density of SIRT1 (161). Another reports in mice have indicated that quercetin attenuates high fat diet-induced atherosclerosis in apolipoprotein E knockout mice by alleviating systemic oxidative stress and also inhibit aortic P47phox by blocking the activation of NADPH oxidase-P47phox membrane translocation (162).

During aging, chronic atherosclerosis promotes ROS production and accumulation which eventually cause mitochondria damage, due to mtDNA damage. OxLDL molecules are immunogenic and are associated with innate immunity to pattern recognition receptors, including scavenger receptors and TLR, and NF-κB, whereas quercetin significantly inhibit NLRP3 inflammatory vesicle activation in the ox-LDL-containing macrophages, thereby attenuating cell lipoatrophy and IL-1β secretion (163). Ex vivo studies have reported that quercetin (25 μM) reduce the HUVEC expression of VCAM-1, where ICAM-1 was significantly enhanced, and then attenuate oxLDL induced endothelial leukocyte adhesion, and effectively modulate the TLR-NF-κB signaling pathway by attenuating the inflammatory process in atherosclerosis (164). Quercetin inhibits the formation of atherosclerotic plaque, and the main mechanism of action is related to the PI3K/AKT-related regulation of caspase-3 and NF-κB activation (165). Moreover, quercetin decrease the mRNA expression of TNF-α and TLR4 in atherosclerotic rats, and the mechanism of action is related to the TLR4-mediated MAPK pathway by reducing the levels of expression of pro-inflammatory cytokines (IL-1β, TNF-α, and IL-10) (166). Dysregulation of TLRs has been reported to increase inflammation and metabolic syndrome which cause the development and progression of atherosclerosis (167).

**Figure 8:**
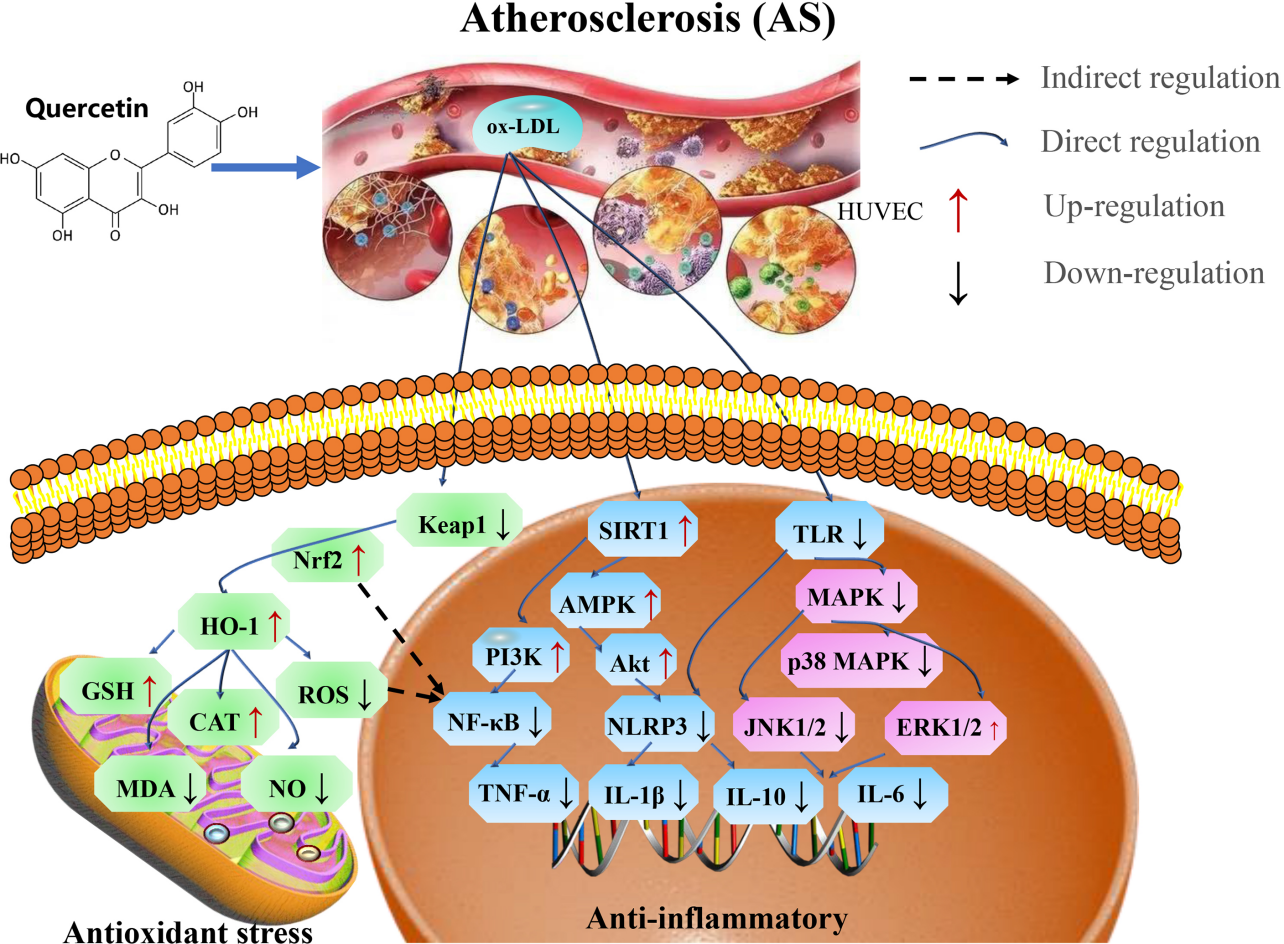
Mechanisms of quercetin on Atherosclerosis. Quercetin is an important target in the treatment of atherosclerosis by preventing endothelial cell damage *via* SIRT1/AMPK/Nrf2 mediated oxidative stress, SIRT1/PI3K/Akt/NF-κB and SIRT1/TLRs/MAPK mediated inflammatory response. TNF-α, tumor necrosis factor-α; AMPK, AMP-activated protein kinase; GSH-Px, glutathione peroxidase; MDA, malondialdehyde; NLRP3, NOD-like receptor protein 3; ox-LDL, oxidized low-density lipoprotein; and TLR4, toll-like receptor 4.

Therefore, quercetin play an important role in the treatment of atherosclerosis by preventing endothelial cell damage *via* SIRT1/AMPK/Nrf2 mediated oxidative stress, SIRT1/PI3K/Akt/NF-κB and SIRT1/TLRs/MAPK mediated inflammatory response (**Figure 8** and **Table 7**).

**Table 7:** Pharmacological function of quercetin on atherosclerosis.

*In vitro* and *in vivo* model	Quercetin Dose	Mechanism	Effect factors	References
male ApoE KO mice	100 mg/kg 16 w	Inhibits Gal-3-NLRP3 signaling pathway	NLRP3↓	(168)
85 patients with CAD	120 mg 2 m	Reduce the transcriptional activity of NF-kB in stable coronary artery disease	TNF-α, IL-1β, IL-10 and IkBα↓	(166)
male ApoE KO mice	25, 50, 100 mg/kg 24 w	Ameliorates atherosclerotic lesions formation	ROS, MDA, NOX4↓ GSH and NADPH↑	(162)
apolipoprotein E-deficient (ApoE-/-) mice and C57BL/6J (C57) mice	20 mg/kg/d 8 w 0.3, 1, or 3 mmol/L 48 h	Attenuates AS by increasing the density of SIRT1	sIcam-1, IL-6, Vcam-1, and ROS ↓ SIRT1 ↑	(161)
high fat diet−induced atherosclerosis in the carotid artery of rats	30 mg/kg/day 2 w	Inhibits inflammatory/oxidative stress responses in AS *via* AMPK/SIRT1/NF-κB pathway	NF-κB, IL−1β, MDA ↓ IL−10, AMPK, SIRT1↑ SOD, CAT, GPX ↑	(160)
endothelial cells	2.5, 5, 10 μM 24 h	Protects against oxLDL-induced endothelial oxidative damage by activating SIRT1	NOX2, NOX4, ROS, NADPH, and ox-LDL ↓ SIRT1, AKT, AMPK ↑	(159)
LDL receptor knockout (−/−) mice	100 μg 30 d	Alleviates oxidative stress *via* diverse pathways, including NF-κB and JAK3	TNF-α, MCP-1, and IL-17α↓ STAT3, SOCS1, PON1 and SRB1↑	(158)
RAW264.7 Macrophage Foam Cells	25, 50 μmol/L 24 h	Regulates MST1-Mediated Autophagy	P53, P21, and P16↓ MST1, LC3-II/I, Beclin1, Bcl-2↑	(157)
male C57BL/6 mice vascular smooth muscle cells (VSMCs)	50, 100 mg/kg 7 w	Suppress inflammation and apoptosis *via* ROS-regulated PI3K/AKT	ROS, Caspase-3 and NF-κB ↓ PI3K/AKT- Bcl-2↑	(165)

h, hours; d, days; w, weeks; m, months; LC3-II/I, microtubule-associated protein light chain 3-II/I; TNF-α, tumor necrosis factor-α; MCP-1, monocyte chemotactic protein-1; STAT3, signal transducer and activator of transcription 3; SOCS1, suppressor of cytokine signaling1; PON1, paraoxonase 1; SRB1, class B scavenger receptor type 1; AMPK, AMP-activated protein kinase; NADPH, nicotinamide adenine dinucleotide phosphate; NOX, NADPH-oxidase; GSH-Px, glutathione peroxidase; MDA, malondialdehyde; NLRP3, NOD-like receptor protein 3; ox-LDL, oxidized low-density lipoprotein; TLR4, toll-like receptor 4; MPO, myeloperoxidase; COX-2, Cyclooxygenase-2; 5-LOX, 5-lipoxygenase; NOS, nitric oxide synthase; CRP, C-reactive protein; VCAM-1, vascular cell adhesion molecule-1; ICAM-1, intercellular adhesion molecule-1; ↓, downregulation; ↑, upregulation.

## 5 Effects of quercetin on diabetes

Diabetes is a metabolic disorder that cause death in human populace worldwide (mostly in elderly people) (169). The International Diabetes Federation reported that people suffering from diabetes equally exhibit neuropathy and neuropathic pain (170). The most common manifestation of diabetic neuropathy is distal symmetric polyneuropathy (DSPN), which affects approximately 30% of diabetic patients with the most relevant clinical manifestations, whereas the incidence of DSPN is approximately 2% per year (171). However, the pathogenesis is unclear, and clinical and epidemiological studies have indicated that oxidative stress and inflammatory processes are important pathological mechanisms in diabetic neuropathy, which is associated with distal symmetric sensorimotor polyneuropathy.

ROS is associated with the development of neuropathy in an experimental diabetes. The findings in streptozotocin (STZ)-injected diabetic rats suggest that oxidative stress cause neurotransmission defects (172–174). Quercetin has been shown to have a beneficial effect on nicotinamide/streptozotocin-induced antidiabetic and antioxidant capacity in Wistar diabetic rats (175, 176), reducing dorsal root ganglion (DRG) neurons damage by oxidative stress (177). Overproduction and accumulation of ROS and reactive carbonyl compounds induces endoplasmic reticulum stress (ERs). Quercetin was found to induce lysosomal defects which triggered ERs and ROS generation, thereby contributing to glioma cell death (29, 178). Quercetin can bypass the GLUT4 translocation insulin regulatory system through the AMPK signaling pathway and its downstream target p38 MAPK, thereby contributing to the correction of insulin resistance (179).An increase AMPK, insulin receptor substrate 1 (IRS-1) and AS160 phosphorylation increase GSK3β under insulin-stimulated conditions (180). Quercetin induces insulin secretion by direct activation of L-type calcium channels in pancreatic beta cells, thus, quercetin interacts with L-type Ca^2+^ channels at a location different from that of Bay K 8644 to increase Ca^2+^ influx, which stimulates insulin secretion (181) and thus, reduce the insulin levels (182). Quercetin improves endothelial function by inhibiting endoplasmic reticulum stress-mediated disruptions leading to the degeneration of islet initiation of the UPR response, and calcium homeostasis, thereby reducing oxidative stress in diabetic rats (183).

SOD is the main antioxidant parameter which prevents neuronal damage (184). Quercetin enhance the expression of antioxidant indices, thereby protects the mitochondrial function by increasing intracellular nicotinamide-adenine dinucleotide (NAD^+^) (185) and may be involved in regulating NF-κB and SIRT1 levels (186, 187). Further mechanisms may be related to the upregulation of SIRT1 activity and protein levels, and its effects on the Akt signaling pathway (188). In diabetic neuropathy, axonal and sensory neuron degeneration pathways are activated, leading to distal axonal lesions. The NAD^+^-dependent deacetylase SIRT1 prevents the activation of these pathways and promotes axonal regeneration (189).

Recent studies have demonstrated that ferroptosis, a newly identified form of regulated cell death characterized by iron-dependent dependence on the overproduction of ROS leading to irreparable lipid peroxidation, is involved in β-cell death leading to reduced insulin secretion. The protective effect of quercetin on mice pancreas occurs partly through the inhibition of hypertrophy (190).

**Figure 9:**
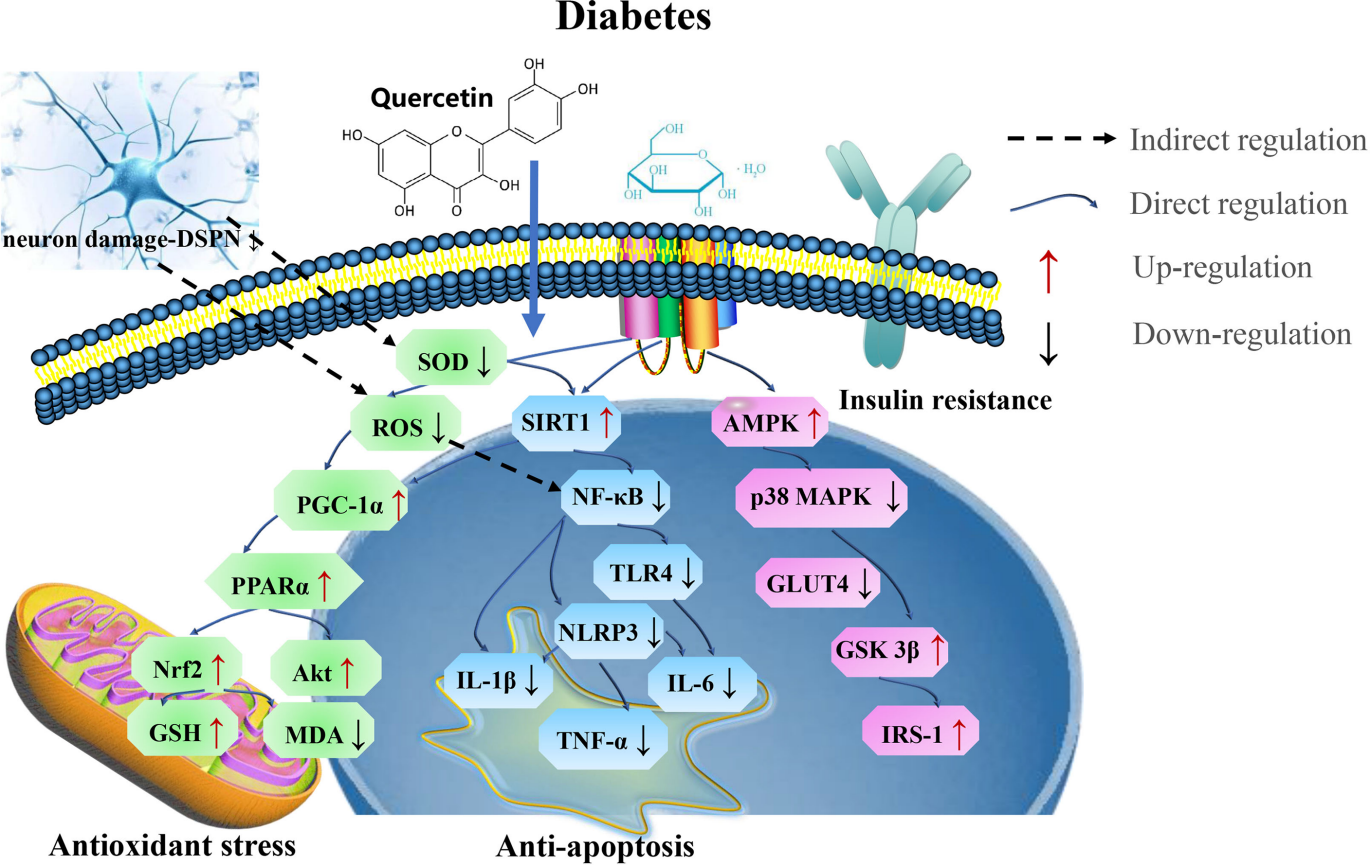
The mechanism of quercetin on diabetes. Quercetin play potential therapeutic roles in treating diabetes by targeting SIRT1 *via* PGC-1α/PPARα/Nrf2 mediated oxidative stress and SIRT1/NF-κB/NLRP3 mediated ferroptosis. SOD, superoxide dismutase; MDA, malondialdehyde; IL-6, interleukin-6; GST, glutathione S-transferases; PGC-1α, proliferator-activated receptor gamma coactivator; IRS-1, insulin receptor substrate-1; GSK3β, glycogen synthase kinase 3 beta.

In general, the application of quercetin at the appropriate dose could serve as a potential therapeutic agent in treating diabetes, by attenuating oxidative stress and ferroptosis (**Figure 9** and **Table 8**).

**Table 8:** Pharmacological functions of quercetin on diabetes.

*In vitro* and *in vivo* model	Quercetin Dose	Mechanism	Effect factors	References
Wistar Diabetic Rats	100 mg/kg 4 w	Antidiabetic Potency, Antioxidant Effects	TC, TG, LDL-C, VLDL, FFA, HOMA-IR, HOMA-IS, and HOMA-β↓ GPx, GST and SOD↑	(176)
Wistar Diabetic Rats	100 mg/kg 4 w	Neurodegenerative diseases	PON2, JNK, TNF-α↓ PGC-1α, MAPKs, CREB, Nrf2 PI3K/Akt ↑	(175)
Male specific-pathogen-free C57BL/6J mice	1.5 g/kg 4 m	Alleviates Ferroptosis	MDA, HOMA↓ GSH, SOD, VDAC2↑	(190)
Male albino Wistar rats	25, 50 mg/kg 1 w	inhibition of endoplasmic reticulum stress-mediated oxidative stress	C/ERB, CHOP, ET-1↓ VEGF↑	(191)
Chinese population	20.9 mg/day 2 m	Protective effect in the development of T2DM	–	(174)
Hyperglycemic Arbor Acre Broilers	10, 25, 50 mg/kg 1 or 2 w	Decrease oxidative stress.	AST, ALT, NO, MDA, MCP-1, IL-6, TNF-α↓ FBG, FINS, SOD, GSH-Px, CAT and PI3K/PKB↑	(182)
Diabetic rats	100mg/kg 15 d	Decrease oxidative stress, inflammation	NF-κB and MDA↓ SIRT1, SOD, CAT↑	(187)
Male Wistar albino rats	100 mg/kg 2 w	regulates insulin metabolism in diabetes	MDA, TNF-α, IL-6↓ GSH↑	(186)
Wistar Diabetic Rats	10 mg/kg 6 d	Improves Glucose and Lipid Metabolism	Akt, SITR1 and GSK-3β↑	(188)
adult male diabetic rats	10, 25, 50 mg/kg 28 d	Decrease oxidative stress, inflammation and apoptosis levels markedly	p65-NF-κB, TNF-α, IL-1β and IL-6↓ SOD, CAT and GPx↑	(181)

h, hours; d, days; w, weeks; m, months; ALT, alanine aminotransferase; AST, aspartate aminotransferase; ALP, alkaline phosphatase; γ-GGT, gamma-glutamyl transferase; LDH, lactate dehydrogenase; CK, creatinine kinase; TC, total cholesterol; TG, triglycerides; HDL, high-density lipoprotein; LDL, low-density lipoprotein; CRE, serum creatinine; BUN, blood urea nitrogen; IRS-1, insulin receptor substrate-1; GSK3β, glycogen synthase kinase 3 beta; NADPH, nicotinamide adenine dinucleotide phosphate; PI3K, phosphoinositide 3-kinase; SOD, superoxide dismutase; CAT, catalase; MDA, malondialdehyde; MCP-1, monocyte chemotactic protein-1; IL-6, interleukin-6; FBG, fasting blood glucose; FINS, fasting insulin; GSH-Px, glutathione peroxidase; FFA, free fatty acids; VLDL, very low-density lipoproteins; HOMA-IR, homeostasis model assessment of insulin resistance; GST, glutathione S-transferases; GPx, glutathione peroxidases; PON2, paraoxonase 2; PGC-1α, proliferator-activated receptor gamma coactivator; VDAC2, voltage-dependent anion channel protein 2; CHOP, C/EBP homologous protein; ET-1, Endothelin-1; VEGF, vascular endothelial growth factor. ↓ downregulation; ↑ upregulation.

## 6 Discussion

Aging is an irreversible physiological process, and the incidence of neurological diseases increases with age (192). Neurodegenerative diseases have been recognized as a manifestation of chronological aging (193). Therefore, it is important to identify suitable anti-aging drugs and explore their therapeutic mechanisms. Oxidative stress, neuroinflammation, apoptosis, autophagy, and mitochondrial dysfunction are significant causative factors related to aging-related diseases (194–197). Recently, numerous studies have reported the protective effects of SIRT1 in aging-related diseases, such as protective effect against neurodegeneration, regulation of oxidative stress, inflammatory response, mitochondrial biogenesis, cell death, and autophagy (17, 18, 198–200). Quercetin is an anti-aging flavonoid, because it possesses antioxidant, anti-apoptotic, anti-inflammatory properties, as well as can actively participate in the improvement of mitochondrial dysfunctions, thereby could be used as a novel therapeutic measure in treating aging-related diseases (29, 59, 96, 196, 201, 202). Therefore, recently researchers focus on investigating the medicinal effects of quercetin and its mechanism in attenuating aging-related diseases.

Several studies have shown that quercetin protects against neurodegenerative diseases by enhancing the mechanism of SIRT1 deacetylase (203, 204). However, low bioavailability and solubility of quercetin limits its clinical application (205). In addition, most of the current studies only focused on quercetin glycosides, however, the functions of the gut micobiota helps to breakdown quercetin into serious biological metabolites such as phloroglucinol, 3,4-dihydroxyphenylacetic acid, 4-hydroxyphenylacetic acid, and 3,4-dihydroxybenzaldehyde (206). Therefore, it is important to examine the potential therapeutic effects of these intestinal metabolites obtained from quercetin.

### 6.1 Quercetin regulates oxidative stress *via* SIRT1

Oxidative stress, mitochondrial dysfunction, inflammatory, and cell autophagy and apoptosis are pathophysiological factors responsible for initiating aging-related diseases (207). There is a correlation between oxidative stress and aging (208). Thus, with chronological aging, ROS production and accumulation from multiple sources increases, whereas the levels of antioxidant enzymes and repair systems (proteasomal degradation) decline. Reports have shown that quercetin improve the concentration antioxidant enzymes and anti-inflammatory cytokines, as well as improve mitochondrial function by targeting SIRT1 activity *via* the SIRT1/AMPK/NF-κB, SIRT1/Keap1/Nrf2/HO-1, and SIRT1/PI3K/Akt pathways (159, 203).

In a rat model, it was indicated that quercetin inhibited ER stress to attenuate oxidative stress-induced apoptosis and prevent the progression of osteoarthritis by activating the SIRT1/AMPK pathway in rat chondrocytes (209), and inhibited oxidative stress responses in diabetic high fat diet-induced atherosclerosis in the carotid artery of rats by modulating AMPK/SIRT1/NF-κB pathway (160). Quercetin inhibited oxidative stress to ameliorate diabetic encephalopathy *via* SIRT1/ER stress pathway in db/db mice (210), furthermore, it inhibited oxidative stress damage to regulate mitophagy and ER stress *via* SIRT1/TMBIM6 (transmembrane BAX inhibitor-1 motif-containing 6) in cardiovascular diseases (211), as well as attenuated collagen-induced oxidative stress in mice arthritis by mediating SIRT1 activation (212). Moreover, Quercetin was found to reduce oxLDL-induced oxidative damage by upregulating AMPK and SIRT1 activity (159); and reduce oxidative damage in the liver and kidney tissues, and NF-kB levels by increasing SIRT1 in diabetes model (187).

### 6.2 Quercetin regulates inflammatory response *via* SIRT1

The attenuation of the inflammatory response is a potential anti-aging strategy. Senescent cell expression is the most pronounced in post-mitotic cells, whereas mitochondria and lysosomes suffer the most significant aging-related alterations in all organelles. Studies have confirmed that SIRT1 activation can inhibit NF-κB, TLRs, and NLRP3 pathways to reduce inflammation. Activation of these SIRT1-dependent signaling pathways by quercetin result in the modulation of the levels and functions of the inflammatory cytokines (213). Quercetin modulates AMPK/SIRT1/NF-κB pathway to inhibit inflammatory responses in diabetic high fat diet-induced atherosclerosis in the carotid artery of rats (160). In addition, quercetin alleviates inflammatory responses by activating SIRT1 and inhibit NF-κB pathway (159), furthermore, it contributes to anti-inflammatory effect by modulating SIRT1 activation *via* AMPK/SIRT1/Nfr2/TNFα pathway (203), as well as counteracts cholesterol-induced activation of the NF-κB pathway in the pancreas and normalized the expression of pro-inflammatory cytokines by increasing SIRTI expression (214), and reduce inflammation response in obesity mice through AMPKα1/SIRT1 pathway (215).

### 6.3 Quercetin regulates mitochondrial function *via* SIRT1

Mitochondrial dysfunction contributes significantly to aging-related diseases (216–218). However, treatment with quercetin could reduce ROS generation and mitigate mitochondrial dysfunction, thereby maintaining the mitochondrial homeostasis and normal function. Quercetin suppressed oxLDL-induced mitochondrial dysfunction and ROS formation by activating SIRT1 and modulating the AMPK/NADPH/AKT pathway (159), and attenuate mitochondrial dysfunction *via* activating AMPK/SIRT1 pathway in osteoarthritis rats (219). Moreover, quercetin mitigate cerebral ischemia reperfusion injury and reduce ROS generation in the mitochondria *via* SIRT1/Nrf2/HO-1 pathway (220).

Quercetin, as SIRT1 agonists, promotes mitochondrial biogenesis thereby attenuates mitochondrial diseases (221), prevented cholesterol-induced mitochondrial bioenergetic dysfunction by upregulating the expression of SIRT1 in the Min6 cells (214), and promotes mitochondrial biogenesis in the brain by activating the transcription of SIRT1 (222, 223). Quercetin as SIRT1/PGC1-α activator was reported to improved cardiac function in aged Mdx/Utrn+/- mice by increasing the protein contents and decrease inflammatory markers NF-κB in the mitochondria (224).

### 6.4 Quercetin regulates autophagy and apoptosis *via* SIRT1

During the occurrence of aging-related diseases, cell become vulnerable to the accumulation of abnormal proteins and damage to the phagocytic lysosomal system, which eventually cause cell death (225). Quercetin-induced autophagy contributes to apoptosis *via* SIRT1/AMPK pathway in lung cancer cells (226), moreover, quercetin inhibits apoptosis and attenuates intervertebral disc degeneration by promoting SIRT1-dependent autophagy (227), as well as improve MI/R-induced cardiomyocyte apoptosis *via* SIRT1/PGC-1α pathway (146), and regulate autophagy and mitochondrial ROS homeostasis *via* Nrf2/PGC-1α/SIRT1 pathway in sodium iodate-induced retinal damage (228). A study identify that quercetin could rescue cardiomyocyte hypoxia by regulating SIRT1/TMBIM6-related mitophagy (211), and reduce renal tubular epithelial cell senescence by activating SIRT1/PINK1/Parkin-mediated mitophagy (229). In addition, other studies have showed that quercetin exerts protective effects against cholesterol-induced apoptosis by improving the expression of SIRTI and inhibits cytochrome c release in Min6 cells (214).

## 7 Conclusions and future perspectives

The pharmacological efficacy of quercetin has shown that it possesses promising therapeutic potentials. In this review, we highlighted the role of quercetin in targeting SIRT1 with the aim of preventing aging-related diseases *via* oxidative stress and inflammation alleviation, as well as the restoration of mitochondrial dysfunction. Therefore, SIRT1 may serve as a potential therapeutic target for the treatment of aging-related diseases.

Recent studies on aging-related diseases have indicated that ferroptosis contributes in the pathogenesis of Alzheimer’s disease (230–232), Parkinson’s disease (233, 234), Huntington’s disease (235), Depression (236–238), Osteoarthritis (239), Myocardial ischemia and reperfusion (MI/R) (240–242), Atherosclerosis (243, 244), and Diabetes (245, 246). Notably, quercetin shows iron-chelating activity and effectively decrease iron deposition in the hearts, kidneys and liver of iron-dextran-overloaded mice (247), and protects bone marrow-derived mesenchymal stem cells from erastin-induced ferroptosis through antioxidant pathway (248). Therefore, quercetin was identified as a ferroptosis inhibitor to alleviate acute kidney injury (249). Moreover, activation of SIRT1 inhibits excess iron-induced ferroptosis of foam cells, thereby providing a novel therapeutic target for atherosclerosis (250), and fisetin attenuated doxorubicin-induced cardiomyopathy by inhibiting ferroptosis *via* the activation of SIRT1/Nrf2 signaling pathway (251). Thus, acting as an intracellular iron chelators, as well as stimulating cellular degradation systems provide novel mechanisms for aging research and may be potential diagnostic biomarkers for aging-related disease in the future (252). The future challenge is to establish models of ferroptosis on disease-specific basis so that the underlying mechanisms of action can be investigated in detail.

Recently, various studies have suggested that extracellular vesicles (EVs) have multiple advantages over currently available drug delivery vehicles, opening new frontiers for modern drug delivery (253–255). Therefore, EVs can be considered for quercetin loading and delivery for treating aging-related disease. In addition, EVs can successfully communicate between cells and organs in the aging microenvironment and during the occurrence of aging-related diseases (256). In particular, neuron-derived EVs can breach the blood-brain barrier (BBB) and become important carriers of signals between cell types in the central nervous system (CNS) (257).

Recent studies have shown that EVs can reduce glutamate expression in the neurons to improve cognitive function and reduce Aβ plaques in AD model mice by activating the SIRT1 pathway (258). The loss of SIRT1 support could accelerated EV production, which carry pathological α-SYN and preferentially target microglia to induce microglial inflammation (259). The release of circulating EVs with oxidative contents alters redox and mitochondrial homeostasis in the brains of rats, suggesting that SIRT1-mediated EVs obtained from different donors may also be promising materials and tools for anti-aging disease therapy.

In this comprehensive review, we have found clues from separate studies and found that quercetin could regulate oxidative stress, inflammatory response, mitochondrial dysfunction, autophagy and apoptosis by activating SIRT1 in aging-related disease. However, there are limited *in vivo* clinical studies on this subject matter, due to limitations such as low bioavailability and solubility of quercetin and disease complexity, therefore, several clinical *in vivo* studies should be carried out to explore the pharmacological effects and the pharmacokinetics of metabolites to establish and identify useful clinical metabolites released from quercetin catabolism by the gut microbiota. Moreover, whether EVs could be considered for quercetin loading and delivery for treating aging-related disease, and whether quercetin could alleviate ferroptosis to protect against aging-related disease by activating SIRT1 requires further studies.

## Author contributions

Conceptualization, ZC and XZ (2nd author); Literature review, YW, GS, YT, XD, and DL; Writing – original draft, ZC and XZ (2nd author); Writing – review & editing, ZC, FA, and XZ (2nd author). All authors have read and agreed to the published version of the manuscript.

## Funding

This study was financially funded by Sichuan Science and Technology Program (2020JDRC0104), the Key Research & Development Plan of the Department of Science and Technology of Tibet Autonomous Region (XZ202101ZY0002N), the Local Projects Guided by the Central Government from Razi County, Tibet Autonomous Region, and the Projects Funded by the Central Government to Guide Local Scientific and Technological Development from Guizhou province (QIANKEZHONGYINDI[2021]4003).

## Conflict of interest

The authors declare that the research was conducted in the absence of any commercial or financial relationships that could be construed as a potential conflict of interest.

## Publisher’s note

All claims expressed in this article are solely those of the authors and do not necessarily represent those of their affiliated organizations, or those of the publisher, the editors and the reviewers. Any product that may be evaluated in this article, or claim that may be made by its manufacturer, is not guaranteed or endorsed by the publisher.
